# Single-cell view and a novel protective macrophage subset in perivascular adipose tissue in T2DM

**DOI:** 10.1186/s11658-024-00668-5

**Published:** 2024-12-03

**Authors:** Jiaxuan Li, Zhenyu Tian, Tongxue Zhang, Jiajia Jin, Xinjie Zhang, Panpan Xie, Haiyan Lin, Junfei Gu, Yingjie Wu, Xiaowei Wang, Shucui Zhang, Xuefang Yan, Dong Guo, Zhe Wang, Qunye Zhang

**Affiliations:** 1 Department of Cardiology, State Key Laboratory for Innovation and Transformation of Luobing Theory, Key Laboratory of Cardiovascular Remodeling and Function Research, Chinese Ministry of Education, Chinese National Health Commission and Chinese Academy of Medical Sciences, Qilu Hospital of Shandong University, Jinan, 250012 China; 2 Department of Geriatrics, Shandong Provincial Hospital Affiliated to Shandong First Medical University, Jinan, 250021 China; 3 Department of Endocrinology, Shandong Provincial Hospital Affiliated to Shandong First Medical University, Jinan, 250021 China; 4 Shandong Provincial Hospital, Shandong Laboratory Animal Center, Science and Technology Innovation Center, Shandong First Medical University and Shandong Academy of Medical Science, Jinan, 250021 China; 5 Key Laboratory of Endocrine Glucose and Lipids Metabolism and Brain Aging, Chinese Ministry of Education, Shandong First Medical University, Jinan, 250021 China; 6 Department of Biology, University College London, London, NW1 2HE UK; 7 Department of Breast and Thyroid Surgery, Liaocheng People’s Hospital, Liaocheng, 252000 China; 8 Department of Neurology, Liaocheng People’s Hospital, Liaocheng, 252000 China

**Keywords:** Diabetes, PVAT, SVF, Single-cell, *Pdpn*^+^ macrophage

## Abstract

**Background:**

Vasculopathy underlies diabetic complications, with perivascular adipose tissue (PVAT) playing crucial roles in its development. However, the changes in the cellular composition and function of PVAT, including the specific cell subsets and mechanisms implicated in type 2 diabetes mellitus (T2DM) vasculopathy, remain unclear.

**Methods:**

To address the above issues, we performed single-cell RNA sequencing on the stromal vascular fraction (SVF) of PVAT from normal and T2DM rats. Then, various bioinformatics tools and functional experiments were used to investigate the characteristic changes in the cellular profile of diabetic PVAT SVF, their implications, and the underlying mechanisms.

**Results:**

Our study reveals the single-cell landscape of the SVF of PVAT, demonstrating its considerable heterogeneity and significant alterations in T2DM, including an enhanced inflammatory response and elevated proportions of macrophages and natural killer (NK) cells. Moreover, macrophages are critical hubs for cross-talk among various cell populations. Notably, we identified a decreased *Pdpn*^+^ macrophage subpopulation in the PVAT of T2DM rats and confirmed this in mice and humans. In vitro and in vivo studies demonstrated that *Pdpn*^+^ macrophages alleviated insulin resistance and modulated adipokine/cytokine expression in adipocytes via the Pla2g2d-DHA/EPA-GPR120 pathway. This subset also enhances the function of vascular endothelial and smooth muscle cells, inhibits vascular inflammation and oxidative stress, and improves vasodilatory function, thereby protecting blood vessels.

**Conclusion:**

*Pdpn*^+^ macrophages exhibit significant vascular protective effects by alleviating insulin resistance and modulating adipokine/cytokine expression in PVAT adipocytes. This macrophage subtype may therefore play pivotal roles in mitigating vascular complications in T2DM. Our findings also underscore the critical role of immune-metabolic cross-talk in maintaining tissue homeostasis.

**Graphical Abstract:**

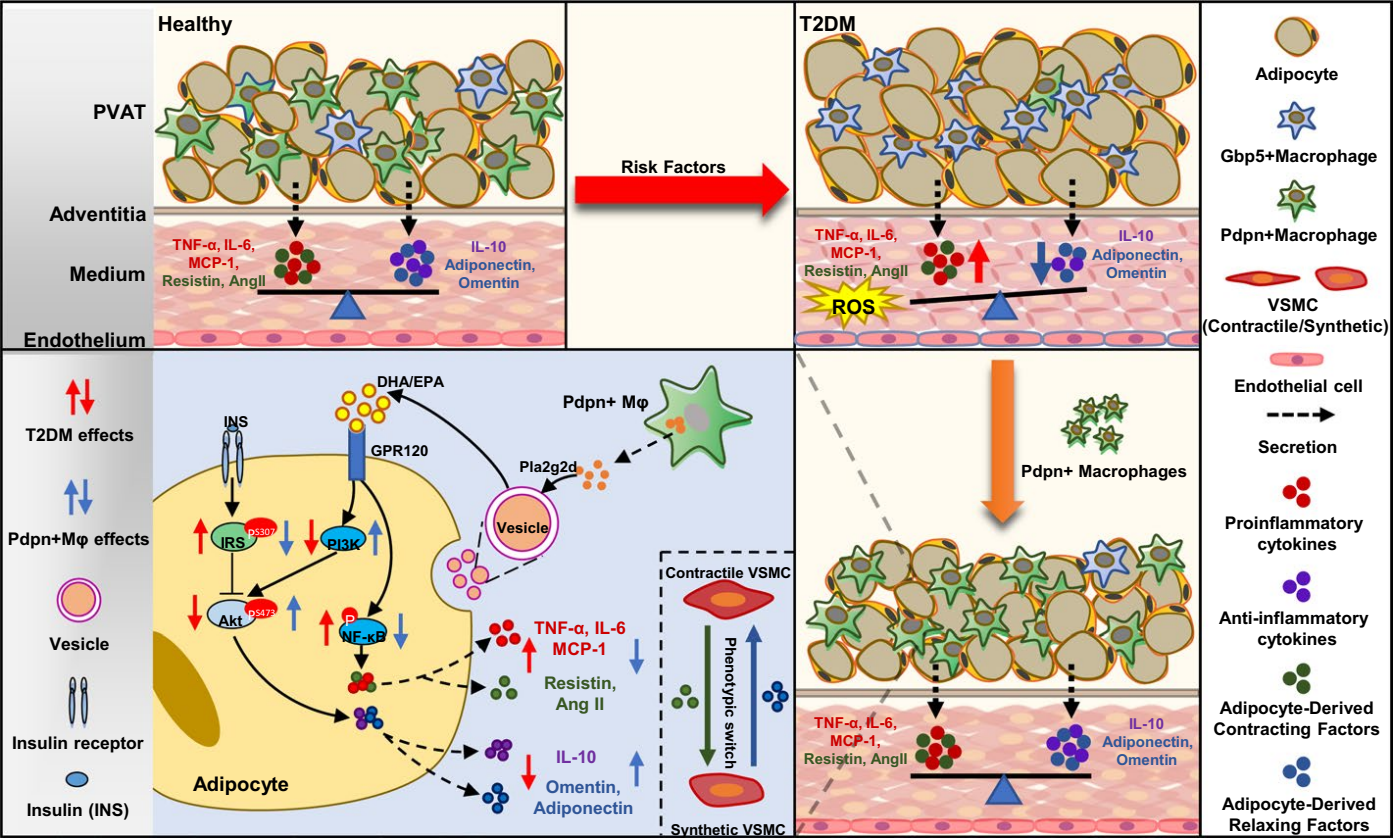

**Supplementary Information:**

The online version contains supplementary material available at 10.1186/s11658-024-00668-5.

## Introduction

In recent years, the incidence of type 2 diabetes mellitus (T2DM) has increased rapidly. Vasculopathy is the main pathological basis for various cardio-cerebrovascular complications of diabetes, which are the major causes of death in diabetic patients [1]. The mechanisms underlying T2DM vasculopathy are intricate and remain incompletely understood. Numerous studies have underscored the important roles of insulin resistance (IR) and inflammation in the vascular complications of diabetes [2, 3]. While much of the research has focused on visceral adipose and vascular tissues, perivascular adipose tissue (PVAT), which is closely related to vasculopathy, has gained increasing attention in recent years [4–6].

PVAT is adjacent to the vascular adventitia and functions not only as a support structure but also as a source of bioactive molecules that regulate vascular functions [7]. Studies have shown that local PVAT in muscle is required for insulin-stimulated vasodilation and microvascular recruitment through adiponectin production [8, 9]. In *db/db* mice, the reduction in adiponectin release from PVAT impaired insulin-dependent Akt signaling, leading to vascular IR [10]. PVAT inflammation exacerbates abnormal vascular remodeling by inducing IR [11]. Some studies have reported that inflammatory factors in the PVAT of diabetic rats inhibit endothelial nitric oxide (NO) production and angiogenesis. However, other studies suggest that the regulatory role of PVAT on the vasculature is bidirectional [11]. PVAT consists of adipocytes and the stromal vascular fraction (SVF), the latter contributing to the complexity of PVAT’s cellular composition and function [12]. Recent studies have shown that PVAT, particularly the immune cells in the SVF, is significantly altered in diabetes and cardiovascular diseases, exhibiting enhanced inflammation and reduced vasodilatory functions [6, 12, 13]. These reports indicate that PVAT plays a central role in the pathogenesis of T2DM vascular complications. However, the mechanisms underlying these effects of PVAT, particularly the cross-talk between immune cells in the SVF and PVAT adipocytes, and their roles in the vascular complications of T2DM, require further investigation.

Macrophages are a predominant cell population in the PVAT SVF [14]. Numerous studies have demonstrated increased macrophage infiltration in the PVAT of diabetic mice [15]. Proinflammatory M1 macrophages in PVAT are commonly believed to trigger diabetic vasculopathy by secreting a variety of cytokines, while anti-inflammatory M2 macrophages function as scavengers, maintaining PVAT homeostasis and normal vascular function [16–18]. Nevertheless, M2 macrophages in PVAT have also been implicated in vascular elastin loss and fibrosis, raising questions about their protective or pathogenic roles [19, 20]. Recent studies have demonstrated that macrophages can be classified into various subpopulations with different functions, whose complexity surpasses the traditional M1 and M2 classification [21]. However, the abnormalities and significance of these macrophage subpopulations of PVAT in T2DM vasculopathy have not been fully elucidated. Based on the current understanding, it is reasonable to hypothesize that the multifaceted roles of PVAT on vascular biology and T2DM-related vasculopathy may be related to the complexity in PVAT macrophage subpopulations and functions. A novel subset of macrophages may exist in the SVF of PVAT, playing a crucial role in the vascular complications of T2DM.

The advent of single-cell RNA sequencing (scRNA-seq) has provided an effective tool for addressing the aforementioned issues. Currently, research on PVAT using scRNA-seq primarily focuses on cardiovascular diseases. A recent study found that *SPP1* + macrophages accumulated in the PVAT surrounding atherosclerotic coronary arteries, promoting fibroadipogenic progenitor cell migration and proliferation through the OPN–CD44/integrin interaction, thereby aggravating coronary PVAT fibrosis, which correlates with coronary stenosis burden [12]. Gu et al. discovered multiple subpopulations of adipose-derived stem cells (ADSCs) in PVAT, some of which participate in vascular regeneration [22]. However, to date, the alterations in PVAT cellular composition and function in T2DM, the cell subsets (especially the macrophage population), and mechanisms implicated in T2DM vasculopathy remain poorly understood.

Motivated by these gaps in knowledge, we conducted the present study. We found that the cellular composition and function of the PVAT SVF are significantly altered in T2DM rats. Notably, a novel protective *Pdpn*^+^ macrophage subpopulation in PVAT is markedly reduced in T2DM. *Pdpn*^+^ macrophage produced Pla2g2d, which exerts vasculoprotective effects via the Pla2g2d-DHA/EPA-GPR120 pathway. Our findings offer new insights into the complex role of PVAT macrophages in the pathogenesis of T2DM vasculopathy and provide a potential target for its therapy.

## Materials and methods

### Human specimens

This study included six patients with T2DM diagnosed according to the American Diabetes Association criteria [23], and six age- and sex-matched non-T2DM individuals with fasting glucose < 126 mg/dL and HbA1c < 6.5%. All participants, aged 38–59 years, were admitted to Liaocheng People’s Hospital (Shandong, China) with body mass indices (BMIs) ranging from 20.1 to 33.6 kg/m^2^. None of the participants had a history of cardiovascular disease, chronic kidney disease, or active infection. Exclusion criteria included current use of corticosteroids or immunosuppressants, pregnant or lactation, and significant hepatic or renal dysfunction. PVAT samples were obtained from the area surrounding the superior thyroid artery of patients with or without diabetes, who were undergoing surgery for thyroid nodules. All participants provided informed consent prior to enrollment. The collection of human samples was approved by the Ethics Committee of Liaocheng People’s Hospital.

### T2DM mice and establishment of a T2DM rat model

Six-week-old male T2DM mice (*db/db* mice) and non-diabetic control mice were obtained from Jackson Laboratory (USA). The mice were housed in a temperature-controlled room with a 12-h light/dark cycle and were fed a standard diet. Diabetic status was confirmed by monitoring body weight, fasting blood glucose (FBG), random blood glucose (RBG), and fasting serum insulin (FINS) levels. Four-week-old male Wistar rats (120–140 g) were obtained from Beijing Vital River Laboratory Animal Technology Co., Ltd. According to previous studies on establishing T2DM rat models [24], the rats were fed a high-fat diet. After 4 weeks, the rats were fasted overnight for 12 h and then administered an intraperitoneal injection of freshly prepared streptozotocin (STZ, Sigma) in citrate buffer (pH 4) at a dosage of 35 mg/kg body weight. FBG was measured using a glucose meter 72 h post-injection. Rats with FBG levels ≥ 11.1 mmol/L were considered successfully modeled for T2DM, while those with FBG levels < 11.1 mmol/L were excluded from the study. During this period, the rat model group continued a high-fat diet, while control rats were fed a standard chow diet and received intraperitoneal injections of citrate buffer alone. FBG, RBG, FINS, glucose tolerance test (GTT), and insulin tolerance test (ITT) were also measured in these rats. Finally, the rats were euthanized, and the thoracic aorta along with its PVAT were dissected for further studies.

### Measurement of FBG, RBG, FINS, GTT, and ITT

FBG and RBG levels in mice and rats were measured with a Bayer Contour Glucose Meter (USA). FINS levels were measured using an ultra-sensitive rat/mouse insulin ELISA kit (Crystal Chem, #90060/90080). The homeostasis model assessment for insulin resistance (HOMA-IR) was calculated as FBG (mmol/L) × FINS (mU/L)/22.5. For the GTT, rats were fasted overnight for 12 h, and baseline blood glucose levels were recorded before an intraperitoneal injection of glucose (2 g/kg body weight [BW]). Glucose levels were measured at 30-min intervals for 120 min. For the insulin tolerance test (ITT), rats were fasted for 6 h, and baseline blood glucose levels were recorded before an intraperitoneal injection of insulin (0.75 IU/kg BW). Glucose levels were measured at 15-min intervals for 60 min.

### PVAT isolation

The thoracic aorta and its PVAT were isolated from rats for scRNA-seq and molecular biology experiments. Rats were anesthetized with an intraperitoneal injection of sodium pentobarbital (60 mg/kg) and then perfused transcardially with saline to remove blood. The thoracic cavity was exposed via a midline sternotomy, and the aorta was excised and rinsed with ice-cold PBS. Under a dissecting microscope, the thoracic aorta and its surrounding PVAT were carefully separated for subsequent experiments. For mice, after euthanasia and saline perfusion, the carotid arteries and their PVAT were isolated as described above for vascular function studies.

### Cell isolation and culture

Adipose tissues were minced into small pieces and digested with 0.2% collagenase (Worthington) at 37 °C for 45 min in a shaker. Digestion was terminated with Dulbecco’s modified Eagle media (DMEM, Gibco) containing 10% fetal bovine serum (FBS, Biological Industries), and the cell suspension was filtered through a 100-μm cell strainer (Solarbio) to remove tissue debris. The stromal vascular fraction (SVF) was resuspended in red blood cell lysis buffer (Solarbio) for 10 min and then filtered through a 40-μm cell strainer. The resulting pellet was resuspended in the appropriate medium for different experiments. For scRNA-seq, the SVF was resuspended in PBS containing 1% bovine serum albumin (BSA). Primary preadipocytes were resuspended in DMEM with 10% newborn calf serum (NCS, Biological Industries), 100 U/mL penicillin, and 100 μg/mL streptomycin (Gibco). Macrophages were resuspended in DMEM with 10% FBS, 100 U/mL penicillin, and 100 μg/mL streptomycin for different experiments.

### Single-cell library preparation and sequencing

To reduce inter-individual variability among samples, we pooled cell suspensions of SVF from ten T2DM rats as the T2DM group sample and from nine control rats as the NC group sample for scRNA-seq analysis. The pooled cell suspensions of SVF (1000 cells/µL, 8 µL per sample), along with reagents, gel beads, and partitioning oil, were loaded onto a 10× chromium chip A to generate a single-cell gel bead-in-emulsion (GEM). The primers provided by the gel bead contained a sequencing primer, barcode, unique molecular identifier (UMI), and poly (dT) sequence. The cells in the GEM were lysed and produced barcoded full-length complementary DNA from polyadenylated mRNA. The scRNA-seq libraries were constructed using a Chromium Single Cell 3’ Reagent Kit v2 (10× Genomics) according to the manufacturer’s protocol. Library quantification and quality were assessed by a Qubit fluorometer (Invitrogen). The libraries were then sequenced on an Illumina HiSeq 2500 platform using paired-end 150 (PE150) mode.

### ScRNA-seq data analysis

Single-cell mRNA expression data were analyzed using the Cell Ranger Single Cell Software Suite (v2.2.0) to perform quality control, sample demultiplexing, barcode processing, and single-cell 3′ gene counting. Sequencing reads were aligned to the *Rattus norvegicus* transcriptome (mRatBN7.2). Barcodes with unique molecular identifier (UMI) counts < 500 were excluded from the analysis. The sequencing saturation was 79.9% for T2DM group and 84.1% for NC group. The sequencing read depth was 487,664,825 for NC group and 620,124,012 for T2DM group. The number of genes detected per cell was 18,694 for NC and 18,051 for T2DM. Downstream analysis was performed using the R package Seurat (version 4.3.0). For quality control, Seurat objects for each sample with the cell-by-gene count matrix were generated using the "CreateSeuratObject" function (min.cells = 3, min.features = 200). After visually assessing data distribution per cell using violin plots, low-quality cells with nCount_RNA < 500 or > 6000, nFeature_RNA < 500, or > 4500, and those with a mitochondrial fraction of > 30% were filtered out.

Subsequently, dimensional reduction, clustering, and visualization were performed. For each sample, data were log-normalized using the “NormalizeData” function. Highly variable genes were identified with the “FindVariableFeatures” function (selection.method = “vst”, nfeatures = 2000), and the count matrix for each sample was scaled using “ScaleData.” Next, dimensional reduction was carried out using “RunPCA” (npcs = 50, verbose = FALSE) followed by “RunTSNE” (reduction = “pca”, dims = 1:20).

DoubletFinder (v2.0.3) was used to predict doublets. The expected number of doublets (nExp) for each dataset was calculated using the formula: total_number_of_cells × 8 × 10^–6^, which translates to an 8% doublet rate for 10,000 recovered cells. The top 20 principal components obtained from principal component analysis (PCA) were used for doublet identification. The proportion of artificially generated doublets (pN) in the merged real-artificial data was set at 0.25. The find.pK function was used to evaluate the appropriateness of the chosen proportion of artificial doublets detected at varying neighborhood sizes (pK) parameter. The parameters used were principal components (PCs) = 1:20, pN = 0.25, and pK = the highest value on the BCmvn plot obtained from find.pK. After doublet filtering, a total of 3.69% and 2.42% of doublets were removed from the negative control (NC) and T2DM groups, respectively.

The gene expression matrices were integrated together using “FindIntegrationAnchors” and “IntegrateData” functions, applying 50 dimensions from Canonical Correlation Analysis (CCA) to correct batch effects between different samples. Dimensionality reduction was performed using “RunPCA” (npcs = 50, verbose = FALSE), followed by RunHarmony (group.by.vars = “sample”) for batch effect correction. After harmonization, clustering was performed with RunTSNE (reduction = “harmony”, dims = 1:20), “FindNeighbors” (reduction = “harmony”, dims = 1:20) and “FindClusters” with a resolution of 0.5 across the first 20 dimensions. To annotate cell types in the integrated dataset, the clusters were matched to major cell types and annotated using the expression of known cell marker genes and the “FindMarkers” function.

### Identification of differentially expressed genes and pathway enrichments

The FindMarkers function in Seurat was used to determine differentially expressed genes (DEGs), with the parameters set to test.use = wilcox, min.percentage (pct) = 0.25, and log_2_ fold change (logfc).threshold = 0.25, and filtering by *P* < 0.05. Genes with a threshold of log_2_fc > 0.5 were considered as marker genes for each cluster and used for annotating cell populations. DEGs were subjected to Gene Ontology (GO) or Kyoto Encyclopedia of Genes and Genomes (KEGG) pathway enrichment analysis. For pathway enrichment analysis, the *P* value cutoff was set as 0.05, and the cutoff of minimum genes was set as 5 [25, 26].

### Intercellular communication analysis

Intercellular communication network was performed using CellChat (v1.1.0), which uses a mass-action model to predict the cell–cell communication probability by integrating gene expression matrix with manually curated databases of signaling ligands, receptors, and cofactors. The centrality score was computed and visualized using the “netAnalysis_computeCentrality” function. Differences in the number and strength of ligand–receptor interactions between different cell populations were compared using “netVisual_diffInteraction.” The communication scores for all ligand–receptor interactions associated with each signaling pathway were calculated using “computeCommunProbPathway.” For CellTalker analysis, putative receptor/ligand interactions were assessed by determining which receptors/ligands were expressed on each cell, using a minimum expression threshold of 1000 (with a maximum expression limit of 20,000) and requiring expression in at least 100 cells within the dataset. After identifying a list of ligands and receptors that met these criteria, putative interaction pairs were identified by matching ligand *x* on cell population *y* with the corresponding receptor z within a given group of cells. Interactions were evaluated separately for NC and T2DM groups and were displayed as circos plots, with ligands colored in blue and receptors in red.

### Fluorescence-activated cell sorting (FACS)-based cell isolation of macrophages in PVAT

The single-cell suspension isolated from SVF in PVAT was diluted to a concentration of 0.5–1 × 10^7^ cells/mL using PBS containing 0.5% BSA and 1% penicillin–streptomycin. To isolate macrophages from PVAT of rats, the anti-Cd68 (1:20) and anti-Pdpn (1:100) antibodies were added. After incubation on ice for 60 min, the cells were incubated in the dark for 30 min with anti-mouse IgG antibody conjugated with Allophycocyanin (APC), and anti-rabbit IgG antibody conjugated with Phycoerythrin (PE). Alternatively, to obtain macrophages from PVAT of mice, the cell suspension was incubated with the following antibodies: anti-mouse Cd68 antibody conjugated with APC and anti-mouse Pdpn antibody conjugated with PE. After incubation on ice in the dark for 30 min, the cells were washed and resuspended in the aforementioned buffer. Then, the cells were sorted using a FACSAria II flow cytometer (BD Biosciences) and the acquired data were analyzed using the FlowJo X software. All antibodies used were listed in Table S2.

### Cell treatments

3T3-L1 cells were procured from the Cell Bank/Stem Cell Bank, Chinese Academy of Science (Shanghai, China). Primary preadipocytes were isolated as described above. The cells were grown to confluence in DMEM containing 10% NCS, 100 U/mL penicillin, and 100 μg/mL streptomycin at 37 °C in a 5% CO_2_ atmosphere. Two days after confluence, the cells were cultured in DMEM containing 0.5 mmol/L IBMX, 1 µmol/L DEX, 10 µg/mL insulin, and 10% FBS for 3 days, followed by 2 days in DMEM containing 10 µg/mL insulin and 10% FBS. Then, the cells were maintained in DMEM containing 10% FBS, with the medium being replaced every 2 days, until 10–14 days after the start of treatment. Mature 3T3-L1 cells were incubated in DMEM containing 1% NCS, 25 mmol/L glucose, and 0.6 nmol/L insulin for 18 h at 37 °C to induce insulin resistance. 3T3-L1 cells or primary preadipocytes were differentiated into adipocytes in the lower compartment of a Transwell system (Transwell with 0.4 μm pore polycarbonate membrane insert). Macrophages were introduced into the upper Transwell compartment and cocultured for 24 h (37 °C, 5% CO_2_). Insulin (100 nM) was added 30 min before the cultures were harvested for the insulin signaling experiments. To isolate extracellular vesicles (ECVs), the mature adipocyte culture supernatant was centrifuged, filtered through 0.22 µm membranes to remove cell debris, and processed with the Total Exosome Isolation Reagent (Invitrogen, USA, 4478359) according to the manufacturer’s instructions. To inhibit ECV secretion, mature adipocytes were treated with 10 μM GW4869 for 30 min. SiRNAs targeting *GPR120* or *Pla2g2d* were synthesized by GenePharma (Shanghai, China). Primary adipocytes or *Pdpn*^+^ macrophages isolated from PVAT were respectively transfected with si-*GPR120* or si-*Pla2g2d *using Lipofectamine RNAiMAX reagent (Life Technologies) according to the manufacturer’s instructions and subjected to 100 μM DHA/EPA treatment. The siRNA sequences used were listed in Table S3.

### Aorta isolation and coculture with macrophages

Aortic vessels were isolated following previously described methods [27]. To analyze the effects of PVAT on isolated arterial rings, macrophages were cocultured with the aortic rings in DMEM-F12 (Gibco) containing 10% FBS, 100 U/mL penicillin, and 100 μg/mL streptomycin at 37 °C with 5% CO_2_. To investigate the influence of adipocytes on isolated arterial rings, fat tissue was removed from the rings, and the arterial ring was cultured in the lower compartment of a Transwell system. Adipocytes and macrophages were cocultured in the upper Transwell compartment using a contact system and then co-incubated with the arterial ring for 24 h at 37 °C with 5% CO_2_.

### Cell transplantation

Primary preadipocytes were isolated from the PVAT of diabetic db/db mice and subsequently induced to differentiate into mature adipocytes in vitro. Then, 1 × 10^6^ adipocytes, along with either *Pdpn*^+^ Mφs or *Pdpn*^*−*^ Mφs previously sorted from PVAT of mice, were mixed in 100 µL Matrigel separately or at a 1:1 ratio. Next, the cell/Matrigel mixture was transplanted around the carotid artery of diabetic *db/db* mice according to the procedure described previously [28]. After 4 weeks of treatment, the carotid arteries from each group of mice were collected for subsequent experiments.

### Western blot analysis

Cells or tissues were washed with cold PBS and lysed in radioimmunoprecipitation assay (RIPA) lysis buffer (Beyotime, Shanghai, China) supplemented with a protease inhibitor cocktail (Beyotime). Total proteins were extracted from the lysate using the Membrane and Cytosol Protein Extraction Kit (Beyotime) according to the manufacturer’s instructions. After quantification by bicinchoninic acid (BCA) assay (Beyotime), the protein extracts were subjected to sodium dodecyl sulfate polyacrylamide gel electrophoresis (SDS–PAGE) electrophoresis and transferred to PVDF membranes (Millipore Sigma). The membranes were blocked with 5% non-fat milk for 1 h at room temperature and incubated with primary antibodies at 4 °C overnight. Next, the membranes were incubated with the corresponding secondary antibodies for 1 h at room temperature. Finally, protein bands were detected using an enhanced chemiluminescence system (Millipore Sigma). Densitometry analysis was performed using ImageJ software. All antibodies used were listed in Table S2.

### Quantitative real-time polymerase chain reaction (qRT-PCR)

Total RNA was isolated using TRIzol (Life Technologies) following the manufacturer’s instructions. PrimeScript RT Master Mix (TaKaRa, Kusatsu, Japan) was used to perform reverse transcription. Quantitative PCR was conducted using TB Green Premix Ex Taq II (TaKaRa). Gapdh was used as the internal standard. The relative mRNA expression levels were quantified using the 2^−ΔΔCt^ method. All the primers used were listed in Table S4.

### Histological analysis

Tissues were fixed overnight in 4% paraformaldehyde, embedded in paraffin, and sectioned transversely into 5-μm-thick sections. Sections were deparaffinized with xylene, rehydrated through a descending ethanol gradient, and then rinsed with distilled water. After antigen retrieval and endogenous peroxidase removal, the sections were blocked in 5% goat serum for 1 h at room temperature and then respectively incubated overnight at 4 °C with the following antibodies: anti-Cd68 (1:100), anti-Gbp5 (1:100), anti-Pdpn (1:100), anti-α-SMA (1:1000), anti-vimentin (1:1000), anti-OPN (1:50), anti-IRS1 (phosphor Ser307, 1:100), anti-Akt (phosphor Ser473, 1:200), anti-GLUT4 (1:200), anti-TNFα (1:100), anti-MCP1 (1:100), or anti-IL10 (1:200). Detection was performed using either secondary antibodies and a DAB peroxidase substrate kit (Beijing Zhongshan Golden Bridge Biotechnology) or secondary antibodies with various fluorescent conjugates (Proteintech), followed by counterstaining with either hematoxylin or 4',6-diamidino-2-phenylindole (DAPI, Beyotime). All the antibodies used were listed in Table S2.

### Reactive oxygen species (ROS) and nitric oxide (NO) assays

The ROS levels were analyzed using a tissue ROS assay kit (GenMed) according to the manufacturer’s instructions. The reaction was carried out at 37 °C for 20 min. ROS levels were calculated on the basis of the fluorescence intensity and protein concentration. NO levels were determined using a NO assay kit (Beyotime) following the manufacturer’s instructions. The absorbance was measured on a microplate reader at 540 nm, and the nitrite concentration was determined using a calibration curve generated from sodium nitrite standards. Nitrite levels were corrected by protein content and expressed as μmol/g protein.

### Glucose consumption assays

Glucose levels of the coculture supernatants were determined using a glucose assay kit (Applygen Technologies, Inc.) according to the manufacturer’s instructions. The absorbance was measured at 550 nm using a microplate reader, and the glucose concentration was calculated against a glucose standard curve. Glucose consumption was calculated as follows: glucose consumption = glucose concentration in the blank group (no cells, only medium) − glucose concentration in each group (model or control groups) [29].

### DHA/EPA assay

Total lipids were extracted with chloroform/methanol and methylesterified in methanol-sulfuric acid solution by heating in a water bath at 80 °C for 30 min. Fatty acid methyl esters were extracted with hexane. Subsequent analysis was performed using gas chromatography mass spectrometry (Shimadzu GCMS-TQ8050 NX) as described previously [30].

### Vascular reactivity assay

Carotid arteries were isolated from euthanized T2DM mice and co-cultured with *Pdpn*^+^ Mφs, *Pdpn*^*−*^ Mφs, or adipocytes for 24 h. The arteries were then mounted on an Automated Multi Wire Myograph System (Model 630MA, DMT, Aarhus, Denmark). Briefly, two tungsten wires (40 μm in diameter) were inserted into the arterial lumen and attached to a force transducer and micrometer. The arteries were immersed in an oxygenated organ bath containing Krebs solution (130 mM NaCl, 4.7 mM KCl, 24.9 mM NaHCO₃, 1.18 mM KH₂PO₄, 1.17 mM MgSO₄, 1.6 mM CaCl₂, 0.026 mM EDTA, and 5.5 mM glucose) and adjusted to the optimal baseline circumference. After a 30-min equilibration period, arterial viability was confirmed using a potassium-rich solution (60 mM). Vascular relaxation was assessed by applying cumulative doses of acetylcholine (ACh, 10^−9^ to 10^−4^ M) to pre-contracted segments treated with norepinephrine (10⁻^5^ M).

### Statistical analysis

Data were presented as means ± standard deviations. The Kolmogorov–Smirnov test was used to evaluate data normality. For normally distributed data, two-tailed Student’s *t*-test and one-way analysis of variance (ANOVA) test with appropriate correction were used for two-group and multiple-group comparisons, respectively. The Mann–Whitney *U* test with Bonferroni correction was used for two-group comparisons in FindMarkers function of Seurat Package. Fisher’s exact test with Benjamini–Hochberg false discovery rate (FDR) multiple-test correction was used for functional enrichment analysis. Statistical analysis was performed using GraphPad Prism v8.1 and R software. A *P* value < 0.05 or an adjusted *P* value < 0.05 was considered statistically significant. All the experiments were repeated for at least five independent replicates.

## Results

### Profound cellular and functional heterogeneity of the SVF of PVAT

**Fig. 1:**
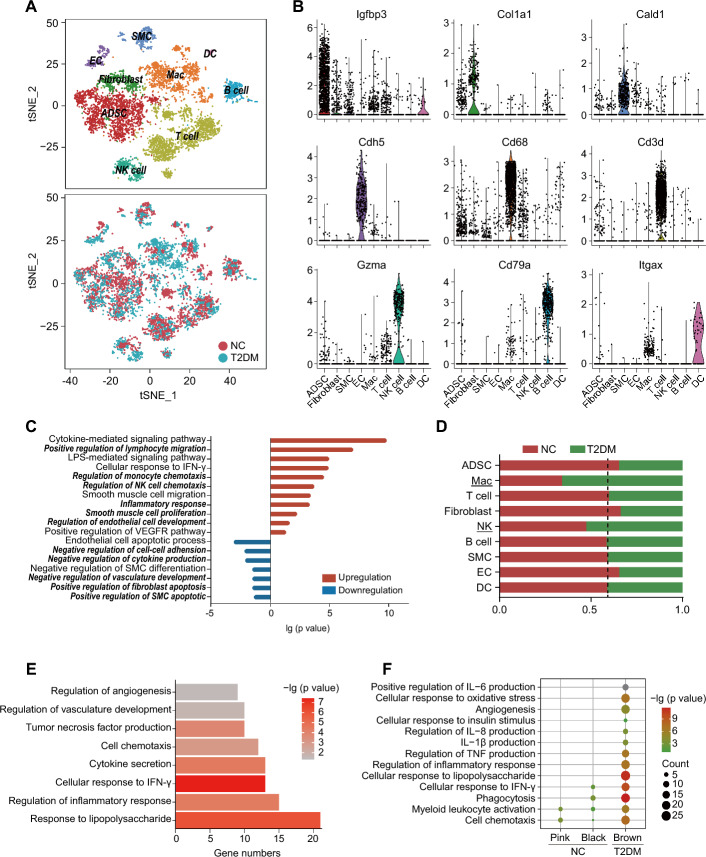
High diversity of cell populations and functions in the SVF of PVAT revealed by scRNA-seq.** A** t-SNE plots showing cell populations in the SVF of PVAT from normal rats (normal control, NC) and rats with T2DM (T2DM). ADSC: adipose-derived stem cell; Mac: macrophage; SMC: smooth muscle cell; EC: endothelial cell; DC: dendritic cell. **B** Violin plots showing the expression levels of marker genes in each cell population of the SVF of PVAT. **C** Functional enrichment analysis of the genes significantly upregulated (red) or downregulated (blue) in the PVAT SVF of T2DM rats. **D** Bar plots depicting the proportion of the number of indicated cell population in the SVF of PVAT data of NC (red) or T2DM (green) rats, compared with the total number of corresponding cell population in the integrated data of the SVF of PVAT. **E** Functional enrichment analysis of genes significantly upregulated in macrophages in the PVAT SVF of T2DM rats. The color bar represents the significance of enrichment analysis. **F** Functional enrichment analysis of the genes in characteristic modules of macrophages in NC (pink and black) and T2DM (brown) rats. The size and color of the bubbles represent the number of genes involved in each function and the significance of enrichment analysis, respectively. Fisher’s exact test with Benjamini–Hochberg false discovery rate (FDR) multiple-test correction was used for the statistical analysis in **C**, **E**, and **F**. Chi-squared test of independence was used for the statistical analysis in **D**

To reveal the cellular composition and function of the SVF of PVAT and its characteristic changes in T2DM, we first established T2DM rat model using a combination of high-fat diet (HFD) feeding and low-dose streptozotocin (STZ) injection. The body weight of T2DM rats decreased significantly after STZ injection, followed by a partial recovery, but remained lower than that of normal rats. Additionally, the changes in FBG, RBG, FINS, HOMA-IR, GTT, and ITT also confirmed the successful establishment of the T2DM rat model (Fig. S1; Table S1). Subsequently, the SVF of the thoracic aortic PVAT of normal and T2DM rats was analyzed by scRNA-seq. Data from 4443 and 3110 cells were obtained from samples of normal and T2DM rats, respectively. The SVF of PVAT in normal and T2DM rats was composed of nine cell types, namely four non-immune cell types including ADSCs, fibroblasts, smooth muscle cells (SMCs) and endothelial cells (ECs) and five immune cell types including macrophages (Macs), T cells, NK cells, B cells and dendritic cells (DCs) (Fig. 1A, B). Notably, ADSCs and fibroblasts showed similar expression profiles of the representative marker genes, as did macrophages and DCs (Fig. S2).

The non-immune cells could be further classified into different subsets. For instance, ADSCs were classified into two major subsets with distinct functions. The ADSC1 subset was involved in more functions than the ADSC2 subset, including the regulation of glycolipid metabolism (Fig. S3A–C). SMCs were also divided into two subsets. The SMC1 subset was primarily associated with the positive regulation of cell adhesion, endothelial cell migration and vasculature development, while the SMC2 subset was mainly involved in lipid metabolism and responses to inflammatory factors (Fig. S3D–F). Similarly, the immune cells could be subdivided into different subsets. For instance, NK cells were categorized into four subsets (Fig. S3G, S3H). *Faslg*^+^ NK cells were associated with antigen processing, presentation, and T-cell activation; *Klra22*^+^ NK cells specifically expressing Klra22 recognized MHC class I molecules to trigger NK and T cell-mediated immune responses; *S100a4*^+^ NK cells were closely linked to glycolysis, oxidative phosphorylation, and ATP synthesis, suggesting increased metabolic activity; and *Tyrobp*^+^ NK cells were primarily related to cell adhesion and T-cell activation (Fig. S3I). These findings highlighted the intricate heterogeneity of cell composition and function in the SVF of PVAT, which was the basis for the complex functions of PVAT.

### Pan-cellular and cell type-specific abnormalities in the SVF of PVAT in T2DM

We found that the expression of a subset of genes was significantly altered across all cell populations in the PVAT SVF of T2DM rats, indicating pan-cellular functional changes. Enrichment analysis revealed that these genes significantly upregulated in the PVAT SVF of T2DM rats were implicated in immune cell migration and chemotaxis, inflammatory responses, and the proliferation and differentiation of endothelial/smooth muscle cells, suggesting an enhancement of these functions in T2DM. Conversely, genes markedly downregulated in T2DM rats were mainly involved in the negative regulation of cell adhesion, vasculature development, and cytokine production, implying a significant suppression of these negative regulatory functions in T2DM (Fig. 1C).

Beyond the pan-cellular functional changes, the number and function of various cell populations in the PVAT SVF of T2DM rats were also significantly altered, exhibiting cell type-specific abnormalities. In T2DM rats, the proportions of macrophages and NK cells significantly increased (Fig. 1D). The genes significantly upregulated in PVAT macrophages of T2DM rats were predominantly related to inflammation and angiogenesis (Fig. 1E). The weighted correlation network analysis (WGCNA) revealed several characteristic gene modules significantly associated with immune cells (adjusted *P* < 0.05, *r* ≥ 0.5) in the SVF of PVAT (Fig. S4A, B). Compared with those in normal rats, the functions of macrophage-specific gene module (brown) in T2DM rats were more complicated and included many inflammatory-related functions and non-immune functions, such as angiogenesis and the cellular response to insulin stimulus (Fig. 1F). Moreover, gene modules associated with T cells, B cells, and DCs also showed an enhancement in inflammation-related functions in the T2DM group (Fig. S4C–E). Within the NK cell populations, *Klra22*^+^ and *S100a4*^+^ NK cell subsets were almost exclusively derived from T2DM samples (Fig. S4F). The genes significantly upregulated in PVAT NK cells of T2DM rats indicated an enhancement in inflammation-related functions and cellular response to hypoxia (Fig. S4G).

### Increased intercellular crosstalk in the SVF of PVAT in T2DM

**Fig. 2:**
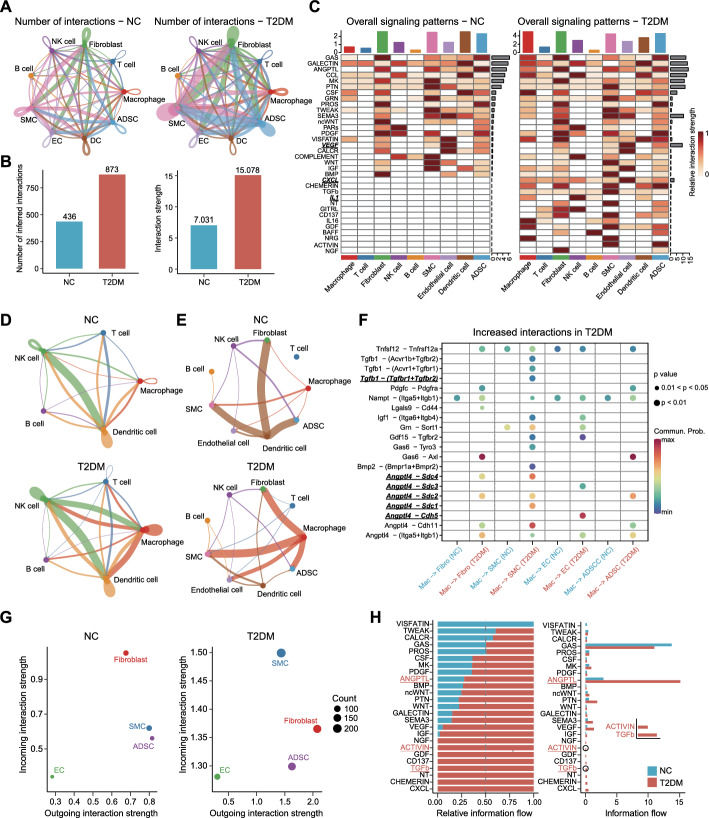
Abnormal intercellular cross-talk in the SVF of PVAT in T2DM rats using CellChat. **A** Number of interactions between various cell populations in the SVF of PVAT. Cells emitting arrows (indicated by arrows) express ligands (receptors). Edge width: interaction numbers. **B** Column plots showing the number (left) or strength (right) of inferred interactions in the SVF of PVAT. Interaction strength: the sum of the communication probabilities of all ligand-receptor pairs. Communication probabilities: the average expression of ligands and receptors in corresponding cell populations. **C** Heatmap of the relative interaction strength of signaling pathways in different cell populations of the SVF of PVAT. Color bar: the relative interaction strength of a signaling pathway. The top (right) bar displays the sum of the relative interaction strength of all (a) signaling pathway(s) in a (all) cell population(s). **D**, **E** Chord diagram illustrating the strength of intercellular communication between immune cells (**D**) and between immune cells and non-immune cells (**E**) in the SVF of PVAT. **F** Dot plot of significantly increased interactions between macrophages and non-immune cells in the SVF of PVAT in T2DM rats. Dot color and size represent the calculated communication probability and *P* value, respectively. **G** Scatter plots of the main senders/sources and receivers/targets of intercellular communications among non-immune cells in the SVF of PVAT. Dot size: the number of inferred interactions associated with each cell population. **H** Bar graph of the relative (left) and overall (right) information flow, which is defined as the total communication probabilities among all pairs of non-immune cell populations in the SVF of PVAT

Intercellular crosstalk is essential for maintaining tissue homeostasis. Our findings demonstrated a notable increase in the number and strength of interactions (ligand–receptor pairs) between different cell types in the PVAT of T2DM rats (Fig. 2A, B; Fig. S5A). The signaling patterns associated with inflammation and angiogenesis, such as VEGF, CXCL, and IL1 signaling, were augmented in T2DM samples compared with normal samples (Fig. 2C). Network centrality analysis showed that macrophages were the most prominent sources of IL1 ligand acting on NK cells, and were also the primary target cells of CXCL family secreted by other cells (Fig. S5B). Our findings indicated a substantial increase in intercellular cross-talk involving macrophages in the PVAT of T2DM rats (Fig. 2C–E). For example, macrophages could act as senders of chemokines and cytokines to other immune cells (e.g., IL16/IL1/CXCL signaling) and as receivers of chemokines secreted by other immune cells (e.g., CCL/CXCL signaling) (Fig. S5C, D). The above results were further confirmed by CellTalker (Fig. S5E–G). Macrophages were found to communicate with Tgfbr1/Tgfbr2-expressing SMCs by secreting Tgfb1, which is known to participate in angiogenesis and the insulin signaling pathway. Macrophages also secreted Angptl4 to interact with Cdh5 expressed on ECs, thereby regulating endothelial cell function. Angptl4 also engaged in cross-talk with syndecans (Sdc1–Sdc4) expressed on non-immune cells, thereby regulating their proliferation and apoptosis (Fig. 2F).

Furthermore, we found that among the non-immune cells in the PVAT of normal rats, fibroblasts were the predominant target cells. However, in T2DM rats, fibroblasts became the most dominant signal exporters, and SMCs became the primary target cells (Fig. 2G). These findings suggested a significant alteration in the regulatory relationships among non-immune cell populations in the PVAT of T2DM rats. Moreover, signaling pathways mediated by ANGPTL, ACTIVIN, and TGFb in non-immune cells of the PVAT SVF were markedly enhanced in T2DM rats (Fig. 2H). These signaling pathways are closely associated with angiogenesis, cell differentiation and migration, and apoptosis. Collectively, these results suggested that the communication between various cell populations was generally enhanced in the PVAT of T2DM rats, with macrophages serving as crucial signaling bridges and hubs.

### Pronounced abnormalities in the proportion and function of macrophages in the SVF of PVAT in T2DM

**Fig. 3:**
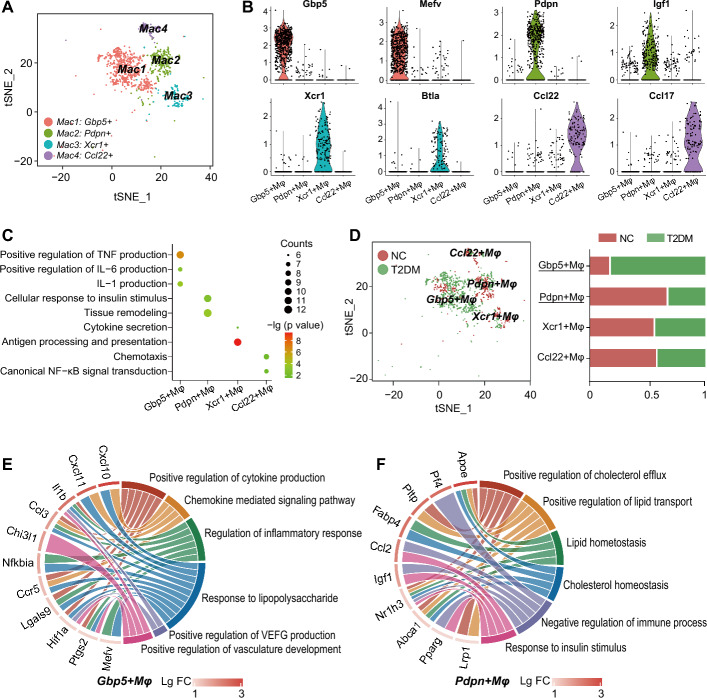
Macrophage subpopulations and abnormal functions in the SVF of PVAT revealed by scRNA-seq. **A** t-SNE plot showing macrophage (Mac) subpopulations in the SVF of PVAT. **B** Violin plots showing the expression levels of marker genes in each macrophage subpopulation of the SVF of PVAT. **C** Functional enrichment analysis of each macrophage subpopulation in the SVF of PVAT. Bubble size and color represent the number of genes involved in corresponding biological process and the significance of enrichment analysis. **D** Left panel: t-SNE plot displaying the subpopulations of macrophages in the SVF of PVAT of normal (red) and T2DM (green) rats. Right panel: bar plots illustrating the proportion of the number of indicated macrophage subpopulation in the SVF of PVAT data of normal (red) or T2DM (green) rats, compared with the total number of the corresponding cell subpopulation in the integrated data of the SVF of PVAT. **E, F** Circos diagram of the relationships between significantly enriched biological processes and the specifically expressed genes in *Gbp5*^+^ Mφs (**E**) or *Pdpn*^+^ Mφs (**F**). FC: fold change in the expression of genes in *Gbp5*^+^ Mφs or *Pdpn*^+^ Mφs compared to other macrophage subpopulations in PVAT SVF. Fisher’s exact test with Benjamini–Hochberg FDR multiple-test correction was used for the statistical analysis in **C**, **E**, and **F**. Chi-squared test of independence was used for the statistical analysis in **D**

The above results demonstrated the most substantial increase in the macrophage populations of the PVAT SVF in T2DM rats. Moreover, the gene modules associated with macrophages showed the strongest correlation with T2DM. Therefore, from a therapeutic perspective, we focused on macrophage populations and performed further analysis. We discovered four macrophage subsets (Fig. 3A). Mac1 subset specifically expressing *Gbp5* and *Mefv* was associated with inflammatory factor production. Mac2 subset characterized by high expression of *Pdpn* and *Igf1* was involved in tissue remodeling and cellular response to insulin. *Xcr1*^+^ macrophages (Mac3) were mainly engaged in antigen processing, presentation, and cytokine secretion. *Ccl22*^+^ macrophages (Mac4) were predominantly related to immune cell chemotaxis and NF-κB signal transduction (Fig. 3B, C). Within the macrophage populations, the proportion of *Gbp5*^+^ macrophages was increased in the T2DM group. This subset was involved in inflammatory responses, vascular endothelial growth factor (VEGF) production and vasculature development (Fig. 3D, E). Conversely, the proportion of *Pdpn*^+^ macrophages was significantly reduced in the PVAT SVF of T2DM rats. This subset was closely related to lipid/cholesterol homeostasis, insulin response, and anti-inflammation processes (Fig. 3D, F). We focused on this macrophage subset in subsequent experiments.

### *Pdpn*^+^ macrophages ameliorate dysfunction and inflammation of arteries in diabetes

**Fig. 4:**
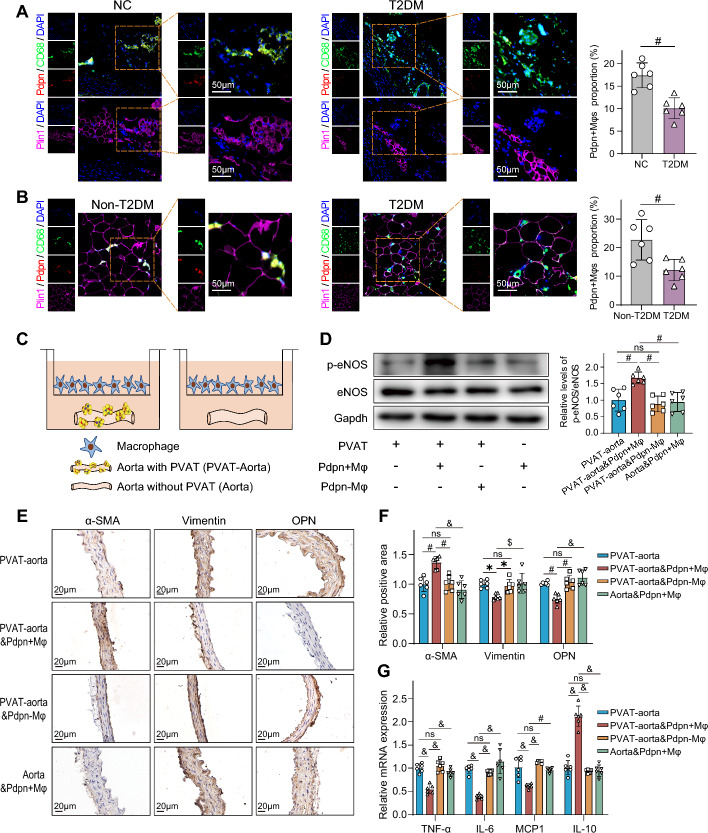
*Pdpn*^+^ macrophages ameliorate arterial abnormalities in diabetes through PVAT. **A, B** Left panel: representative immunofluorescence images of *Cd68*^+^*Pdpn*^+^ Mφs in PVAT. Right panel: comparison of the proportions of *Cd68*^+^*Pdpn*^+^ Mφs to *Cd68*^+^ Mφs in PVAT of normal (negative control, NC) and T2DM rats (**A**) or non-T2DM individuals (Non-T2DM) and patients with T2DM (**B**). For **A**, CD68 (green), Pdpn (red), and the adipocyte marker perilipin 1 (Plin1, pink) were stained on two consecutive paraffin sections. The first section (top) was stained for CD68 (green) and Pdpn (red), while the second section (bottom) was stained for perilipin 1 (Plin1, pink). **C** Schematic diagram of the coculture system of macrophages and aorta with PVAT (PVAT-aorta) or aorta without PVAT (Aorta) isolated from T2DM rats. **D** Left panel: representative western blots showing endothelial nitric oxide synthase (eNOS) and phosphorylated eNOS (p-eNOS) in the aortae of each group. Right panel: comparison of the p-eNOS/eNOS level in each group. PVAT-aorta: aorta with PVAT; PVAT-aorta and *Pdpn* + Mφ (PVAT-aorta and *Pdpn*-Mφ): aorta with PVAT cocultured with *Pdpn*^+^ Mφs (*Pdpn*^−^ Mφs); Aorta and *Pdpn* + Mφ: aorta without PVAT cocultured with *Pdpn*^+^ Mφs. **E, F** Representative immunohistochemistry images of VSMC phenotypic transformation markers in the aortas of each group (**E**), and their quantitative comparison (**F**). The groups are described in **D**. **G** Comparison of mRNA expression of proinflammatory and anti-inflammatory cytokines in the aortas of each group. The groups are described in **D**. The data are presented as the means ± SDs. A two-tailed Student’s t test was used for the statistical analysis in **A** and **B**. One-way ANOVA was used for the statistical analysis in **D**, **F**, and **G**. *P < 0.05; $*P* < 0.01; #*P* < 0.001; &*P* < 0.0001; ns: no significance

Before proceeding with functional studies, we verified the decrease in *Pdpn*^+^ Mφs noted previously. Immunofluorescence showed that the proportion of *Pdpn*^+^ Mφs was significantly decreased in the PVAT of T2DM rats (Fig. 4A). Moreover, a significant decrease in the proportion of *Pdpn*^+^ Mφs was also observed in the PVAT of patients with T2DM (Fig. 4B) and mice (Fig. S6A), highlighting the ubiquity and clinical relevance of the reduction of *Pdpn*^+^ Mφs. The basic physiological characteristics of the mice and the clinical data of the human subjects are presented (Table S1; Fig. S6B–D). Additionally, our results demonstrated that *Pdpn*^+^ Mφs were not detected in the subcutaneous adipose tissue (SAT) and visceral adipose tissue (VAT) isolated from rats, suggesting their PVAT specificity (Fig. S6E). We found that *Pdpn*^+^ Mφs significantly increased the phosphorylation level of endothelial nitric oxide synthase (p-eNOS/eNOS) in the aorta with PVAT (PVAT-aorta) isolated from T2DM rats, indicating enhanced eNOS activity (Fig. 4C, D). Moreover, *Pdpn*^+^ Mφs significantly upregulated the expression of α-SMA, a contractile vascular smooth muscle cell (VSMC) marker and inhibited the expression of synthetic VSMC markers (vimentin and OPN) in PVAT-aorta isolated from T2DM rats (Fig. 4E, F). These results suggested that *Pdpn*^+^ Mφs significantly promoted the phenotypic conversion of VSMCs to a contractile phenotype. Furthermore, *Pdpn*^+^ Mφs significantly reduced the expression of proinflammatory cytokines (TNF-α, IL-6, and MCP-1) and increased the expression of the anti-inflammatory cytokine (IL-10) in PVAT-aorta isolated from T2DM rats (Fig. 4G). However, when PVAT was removed, the above-mentioned effects of *Pdpn*^+^ Mφs were almost completely lost, indicating that the protective effects of *Pdpn*^+^ Mφs were PVAT-dependent. In contrast, when cocultured with aortae with PVAT, *Pdpn*^*−*^ Mφs had none of the above-mentioned protective effects of *Pdpn*^+^ Mφs (Fig. 4D–G). These results all suggested that *Cd68*^+^*Pdpn*^+^ macrophages ameliorated the dysfunction and inflammation of arteries in diabetes through PVAT.

### The protective effects of *Pdpn*^+^ macrophages on diabetic arteries are dependent on adipocytes in PVAT

**Fig. 5:**
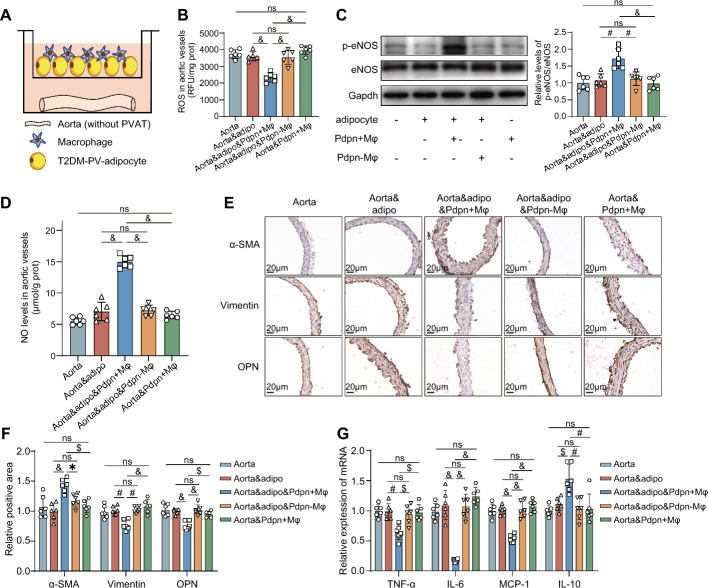
The protective effects of *Pdpn*^+^ macrophages on diabetic aortae are dependent on adipocytes of PVAT. **A** Schematic diagram of the coculture system of aortae without PVAT isolated from T2DM rats, macrophages derived from the PVAT of normal rats, and adipocytes derived from the PVAT of T2DM rats (T2DM-PV-adipocyte). **B** Comparison of ROS levels in the aortae of each group. Aorta: aorta without PVAT of T2DM rats; Aorta&adipo: aorta cocultured with T2DM-PV-adipocytes; Aorta&adipo& *Pdpn* + Mφ (Aorta&adipo&*Pdpn*-Mφ): aorta cocultured with T2DM-PV-adipocytes and *Pdpn*^+^ Mφs (or *Pdpn*^−^ Mφs); Aorta&*Pdpn* + Mφ: aorta cocultured with *Pdpn*^+^ Mφs. **C** Left panel: representative Western blots showing eNOS and p-eNOS in the aortae of each group. Right panel: comparison of p-eNOS/eNOS level in each group. The groups are described in **B**. **D** Comparison of nitric oxide (NO) levels in the aortae of each group. The groups are described in **B**. **E, F** Representative immunohistochemistry images of VSMC phenotypic transformation markers in the aortae of each group (**E**), and their quantitative comparison (**F**). The groups are described in **B**. **G** Comparison of mRNA expression of proinflammatory and anti-inflammatory cytokines in the aortas of each group. The groups are described in **B**. Data are presented as the means ± standard deviations (SDs). One-way ANOVA was used for the statistical analysis. *P < 0.05; $*P* < 0.01; #*P* < 0.001; &*P* < 0.0001; ns: no significance

As the most abundant cells in PVAT, adipocytes play key roles in vascular lesions of T2DM. To elucidate the mechanisms by which *Pdpn*^+^ Mφs ameliorated the dysfunction and inflammation of diabetic aortae via PVAT, the aortae without PVAT was isolated from T2DM rats and cocultured with *Pdpn*^+^ Mφs derived from the PVAT of normal rats and/or adipocytes derived from the PVAT of T2DM rats (T2DM-PV-adipocytes) (Fig. 5A). The results demonstrated that when cocultured with diabetic aortae and T2DM-PV-adipocytes (Aorta&adipo), *Pdpn*^+^ Mφs significantly reduced the level of reactive oxygen species (ROS) and alleviated oxidative stress in diabetic aortae (Fig. 5B). Moreover, *Pdpn*^+^ Mφs notably increased the levels of phosphorylated eNOS (p-eNOS/eNOS) and nitric oxide (NO), indicating that *Pdpn*^+^ Mφs significantly attenuated the dysfunction of the NO pathway in the diabetic aortae cocultured with T2DM-PV-adipocytes (Aorta&adipo&*Pdpn +* Mφ) (Fig. 5C, D). *Pdpn*^+^ Mφs also significantly promoted the conversion of VSMCs to the contractile phenotype (Fig. 5E, F). Additionally, *Pdpn*^+^ Mφs markedly decreased the expression of proinflammatory cytokines (TNF-α, IL-6, and MCP-1) and upregulated the expression of IL-10 in diabetic aortae, thereby exerting anti-inflammatory effects (Fig. 5G). However, the aforementioned effects of *Pdpn*^+^ Mφs disappeared when *Pdpn*^+^ Mφs were cocultured only with diabetic aortae (Aorta&*Pdpn +* Mφ), namely, in the absence of T2DM-PV-adipocytes. Moreover, even when cocultured with T2DM-PV-adipocytes and diabetic aortae, *Pdpn*^*−*^ Mφs failed to replicate the protective effects of *Pdpn*^+^ Mφs, as described above. Similarly, the above-mentioned indicators did not significantly change when aortae without PVAT, isolated from T2DM rats, were cocultured only with T2DM-PV-adipocytes (Fig. 5B–G). All these results suggested that the protective effects of *Pdpn*^+^ Mφs on diabetic arteries were dependent on adipocytes in PVAT.

### *Pdpn*^+^ macrophages ameliorate insulin resistance and remodel adipokine/cytokine expression in adipocytes

**Fig. 6:**
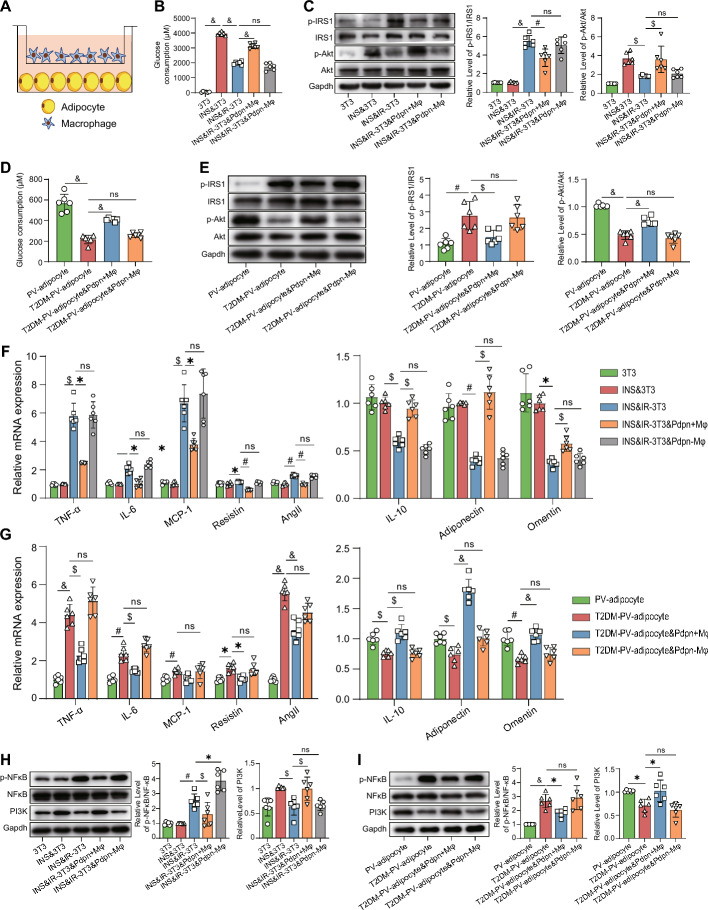
*Pdpn*^+^ macrophages mitigate insulin resistance and remodel adipokine/cytokine expression in 3T3-L1 cells and PV adipocytes. **A** Schematic diagram of the coculture system of macrophages and adipocytes. **B** Comparison of the glucose consumption of 3T3-L1 cells in each group. 3T3: untreated 3T3-L1 cells; INS and 3T3: 3T3-L1 cells treated with 100 nM insulin; INS and IR-3T3: insulin resistant 3T3-L1 cells treated with 100 nM insulin; INS and IR-3T3 and *Pdpn* + Mφ (INS and IR-3T3 and *Pdpn*-Mφ): insulin resistant 3T3-L1 cells treated with 100 nM insulin were cocultured with *Pdpn*^+^ Mφs (or *Pdpn*^−^ Mφs). **C** Left panel: representative western blots of phosphorylated IRS1 (Ser307, p-IRS1), total IRS1 (IRS1), phosphorylated AKT (Ser473, p-Akt), and total AKT (Akt) in 3T3-L1 cells of different groups. Right panel: comparison of p-IRS1/IRS1 and p-Akt/Akt levels in each group. The groups are described in **B**. **D** Comparison of the glucose consumption in PVAT-derived adipocytes (PV adipocyte) treated with 100 nM insulin in each group. PV adipocyte: primary adipocytes derived from PVAT of normal rats; T2DM PV adipocyte: primary adipocytes derived from PVAT of T2DM rats; T2DM PV adipocyte and *Pdpn* + Mφ (T2DM PV adipocyte and *Pdpn*-Mφ): T2DM-PV-adipocytes cocultured with *Pdpn*^+^ Mφs (or *Pdpn*^−^ Mφs). **E** Left panel: representative Western blots of phosphorylated IRS1 (Ser307, p-IRS1), total IRS1 (IRS1), phosphorylated AKT (Ser473, p-Akt), and total AKT (Akt) in PV-adipocytes of different groups. Right panel: comparison of p-IRS1/IRS1 and p-Akt/Akt levels in each group. The groups are described in **D**. **F, G** Comparison of mRNA expression of adipokines/cytokines in 3T3-L1 cells (**F**) or PV-adipocytes (**G**) of different groups. The groups are described in **B** and **D**, respectively. **H, I** Left panel: representative western blots of phosphorylated NF-κB (p-NFκB), total NF-κB (NFκB), and PI3K in 3T3-L1 cells (**H**) or PV adipocytes (**I**) of different groups. Right panel: comparison of the levels of p-NFκB/NFκB and PI3K expression in each group. The groups are described in **B** and **D**, respectively. Data are presented as the means ± SDs. One-way ANOVA was used for the statistical analysis. *P < 0.05; $*P* < 0.01; #*P* < 0.001; &*P* < 0.0001; ns: no significance

Insulin resistance in adipocytes is a key mechanism of vascular injury in diabetes. Our scRNA-seq analysis suggested that *Pdpn*^+^ Mφs were closely related to insulin resistance. Moreover, our functional studies demonstrated that the protective effects of *Pdpn*^+^ Mφs on diabetic arteries were dependent on adipocyte. Therefore, we investigated the effects of *Pdpn*^+^ Mφs on the insulin resistance in adipocytes by coculturing *Pdpn*^+^ Mφs with adipocytes (Fig. 6A). The results showed that insulin significantly induced glucose consumption in 3T3-L1 cells, while this effect was markedly diminished in insulin-resistant 3T3-L1 cells (IR-3T3). After coculture, *Pdpn*^+^ Mφs significantly heightened glucose consumption in IR-3T3 cells treated with insulin (Fig. 6B). Mechanistically, we found that *Pdpn*^+^ Mφs significantly decreased the level of p-IRS1 (Ser307)/IRS1 and increased the level of p-Akt (Ser473)/Akt in IR-3T3 cells, thereby substantially enhancing the insulin signaling pathway and ameliorating insulin resistance in adipocytes (Fig. 6C). However, *Pdpn*^*−*^ Mφs did not exhibit the aforementioned effects of *Pdpn*^+^ Mφs (Fig. 6B, C). To confirm the findings from 3T3-L1 cells, we further investigated the effects of *Pdpn*^+^ Mφs on insulin resistance in insulin-treated primary adipocytes isolated from PVAT (PV-adipocytes) of rats, as previously described [31, 32]. The results were consistent with those obtained from 3T3-L1 cells, namely, *Pdpn*^+^ Mφs significantly enhanced glucose consumption and insulin signaling pathway and ameliorated insulin resistance in PV-adipocytes of T2DM rats (T2DM-PV-adipocyte), while *Pdpn*^*−*^ Mφs also did not show the above-mentioned beneficial effects of *Pdpn*^+^ Mφs (Fig. 6D, E).

Adipocytes in PVAT are known to secrete many adipokines and cytokines, playing key roles in vascular function. We found that the expression of proinflammatory cytokines (TNF-α, IL-6, and MCP-1) and vasocontractile adipokines (resistin and AngII/AGT) was significantly increased in insulin-treated IR-3T3 cells (INS&IR-3T3) and insulin-treated T2DM-PV-adipocytes, whereas the expression of anti-inflammatory cytokine (IL-10) and vasorelaxant adipokines (adiponectin and omentin) was significantly decreased (Fig. 6F, G). Moreover, compared with normal adipocytes, the level of p-NFκB/NFκB was significantly enhanced in INS and IR-3T3 cells and T2DM-PV-adipocytes, while the expression of PI3K was significantly suppressed. These findings suggested that the NF-κB signaling pathway, mediating proinflammatory factor production, was significantly activated, whereas the activation of the PI3K pathway, promoting adiponectin expression, was inhibited (Fig. 6H, I). When *Pdpn*^+^ Mφs were cocultured with insulin-treated IR-3T3 cells or insulin-treated T2DM-PV-adipocytes, they significantly attenuated the above-mentioned abnormalities in cytokine and vasoactive factor expression, as well as the NF-κB and PI3K pathways, thus reducing the dysfunction of adipocytes induced by insulin resistance. *Pdpn*^*−*^ Mφs, however, did not have these protective effects of *Pdpn*^+^ Mφs (Fig. 6F–I).

### *Pdpn*^+^ macrophages regulate adipocyte function through the Pla2g2d-DHA/EPA-GPR120 pathway

**Fig. 7:**
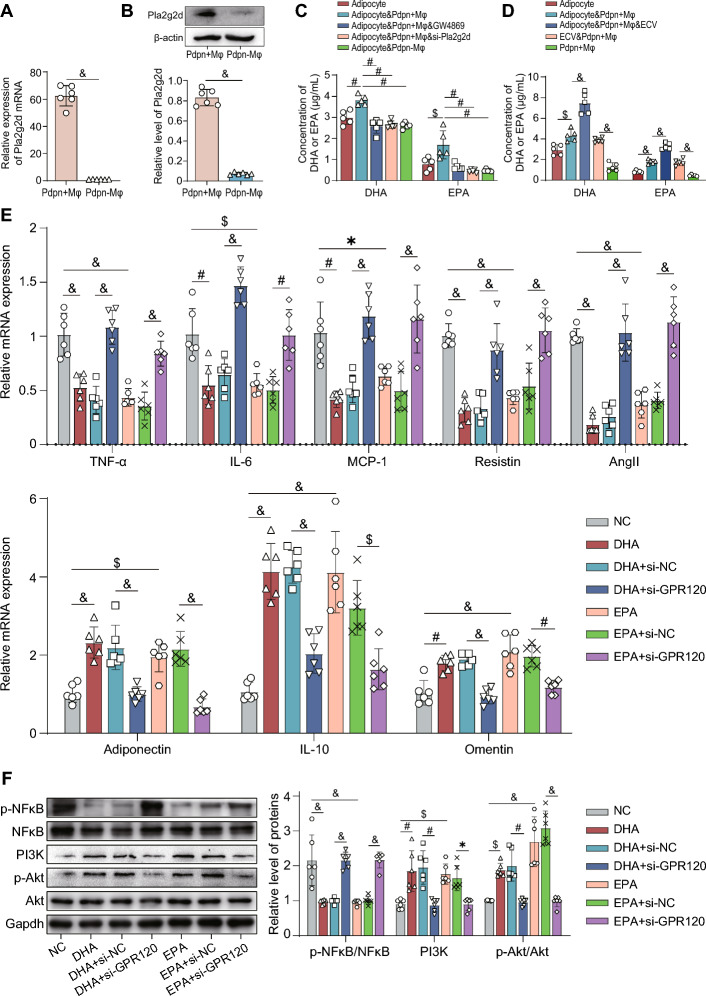
*Pdpn*^+^ macrophages regulate adipocyte function through the Pla2g2d-DHA/EPA-GPR120 signaling pathway. **A, B** Relative expression levels of Pla2g2d mRNA (**A**) and protein (**B**) in *Pdpn*^+^ Mφs and *Pdpn*^−^ Mφs derived from PVAT detected by qPCR and western blot, respectively. **C** Concentrations of DHA/EPA in the culture supernatant of each group. Adipocyte: T2DM PVAT adipocytes without any treatment; adipocyte and *Pdpn* + Mφ (adipocyte and *Pdpn*-Mφ): T2DM PVAT adipocytes cocultured with *Pdpn*^+^ Mφs (or *Pdpn*^−^ Mφs); adipocyte and *Pdpn* + Mφ and GW4869: T2DM PVAT adipocytes treated with GW4869 were cocultured with *Pdpn*^+^ Mφs. Adipocyte and *Pdpn* + Mφ and si-Pla2g2d: T2DM-PVAT-adipocytes were cocultured with *Pdpn*^+^ Mφs treated with si-*Pla2g2d*. **D** Concentrations of DHA/EPA in the culture supernatant of each group. Adipocyte: T2DM PVAT adipocytes without any treatment; adipocyte and *Pdpn* + Mφ: T2DM-PVAT-adipocytes cocultured with *Pdpn*^+^ Mφs; adipocyte and *Pdpn* + Mφ and ECV: T2DM-PVAT-adipocytes and ECV cocultured with *Pdpn*^+^ Mφs. ECV and *Pdpn* + Mφ: ECV cocultured with *Pdpn*^+^ Mφs. *Pdpn* + Mφ: *Pdpn*^+^ Mφs without any treatment. **E** Comparison of relative mRNA expression of proinflammatory/vasocontractile and anti-inflammatory/vasorelaxant factors in T2DM PVAT adipocytes with different treatments. NC: T2DM-PVAT-adipocytes without treatments; DHA (EPA): T2DM PVAT adipocytes treated with DHA (or EPA); DHA + si-NC (EPA + si-NC): T2DM PVAT adipocytes treated with DHA (or EPA) and the negative control sequence of siRNA (si-NC); DHA + si-*GPR120* (EPA + si-*GPR120*): T2DM PVAT adipocytes treated with DHA (or EPA) and si-*GPR120*. **F** Left panel: representative images of western blot of phosphorylated NF-κB (p-NFκB), NFκB, PI3K, phosphorylated AKT (p-Akt) and AKT in T2DM PVAT adipocytes with different treatments. Right panel: the quantitative comparison of p-NFκB/NFκB level, PI3K expression and p-Akt/Akt level. The groups are described in **E**. The data are presented as the means ± SDs. Two-tailed Student’s *t*-test was used for the statistical analysis in **A** and **B**. One-way ANOVA was used for the statistical analysis in **C–F**. *P < 0.05; $*P* < 0.01; #*P* < 0.001; &*P* < 0.0001

Our findings demonstrated that *Pdpn*^+^ macrophages, rather than *Pdpn*^*−*^ macrophages, ameliorated vascular injury in T2DM in an adipocyte-dependent manner, suggesting that Pdpn might play a key role in the vasoprotective effects of *Pdpn*^+^ macrophages by alleviating insulin resistance in adipocytes. Our scRNA-seq data showed a relatively specific expression of phospholipase A2 IIG IID (Pla2g2d) in *Pdpn*^+^ macrophages (Fig. S6F). Western blot and qPCR results further confirmed the presence of *Pla2g2d* in *Pdpn*^+^ macrophages, rather than in *Pdpn*^*−*^ macrophages, derived from PVAT (Fig. 7A, B). *Pla2g2d* has been reported to hydrolyze membrane phospholipids of extracellular vesicles, producing ω3 polyunsaturated fatty acids (ω3 PUFA) such as DHA and EPA, which exert anti-inflammatory and metabolic regulatory functions [33, 34]. Our results indicated that coculturing *Pdpn*^+^ macrophages with adipocytes significantly increased the levels of DHA and EPA in the culture supernatant. However, when the extracellular vesicles (ECVs) secretion of adipocytes was suppressed using GW4869, or the expression of *Pla2g2d* in *Pdpn*^+^ macrophages was inhibited by siRNA, DHA/EPA levels in the culture supernatant were significantly decreased (Fig. 7C; Fig. S6G). Additionally, ECVs secreted by adipocytes further significantly increased the levels DHA and EPA in the co-culture system of *Pdpn*^+^ macrophages and adipocytes. Replacing adipocytes with their secreted ECV did not affect the levels of DHA and EPA in the culture supernatant (Fig. 7D**)**. It is well known that DHA and EPA can bind to the *GPR120* receptor and regulate its downstream pathways, including PI3K/Akt pathway activation and NF-κB pathway inhibition [35]. Therefore, we speculated that *Pdpn*^+^ macrophages also regulate adipocyte function through the Pla2g2d-DHA/EPA-GPR120 pathway. Our results demonstrated that in primary diabetic adipocytes, DHA/EPA significantly downregulated the expression of proinflammatory cytokines (TNF-α, IL-6, and MCP-1) and vasocontractile adipokines (resistin and AngII/AGT), while upregulating the expression of anti-inflammatory cytokine (IL-10) and vasorelaxant adipokines (adiponectin and omentin) (Fig. 7E). Mechanically, DHA/EPA significantly downregulated the p-NFκB/NFκB level, and upregulated PI3K expression and the p-Akt/Akt level (Fig. 7F). However, when *GPR120* was knocked down in adipocytes using si-*GPR120* (Fig. S6H), the aforementioned effects of DHA/EPA were abolished (Fig. 7E, F). These results confirmed our speculation that *Pdpn*^+^ macrophages regulated adipocyte function through the Pla2g2d-DHA/EPA-GPR120 pathway.

### *Pdpn*^+^ macrophages significantly improve vasculopathy in diabetic mice

**Fig. 8:**
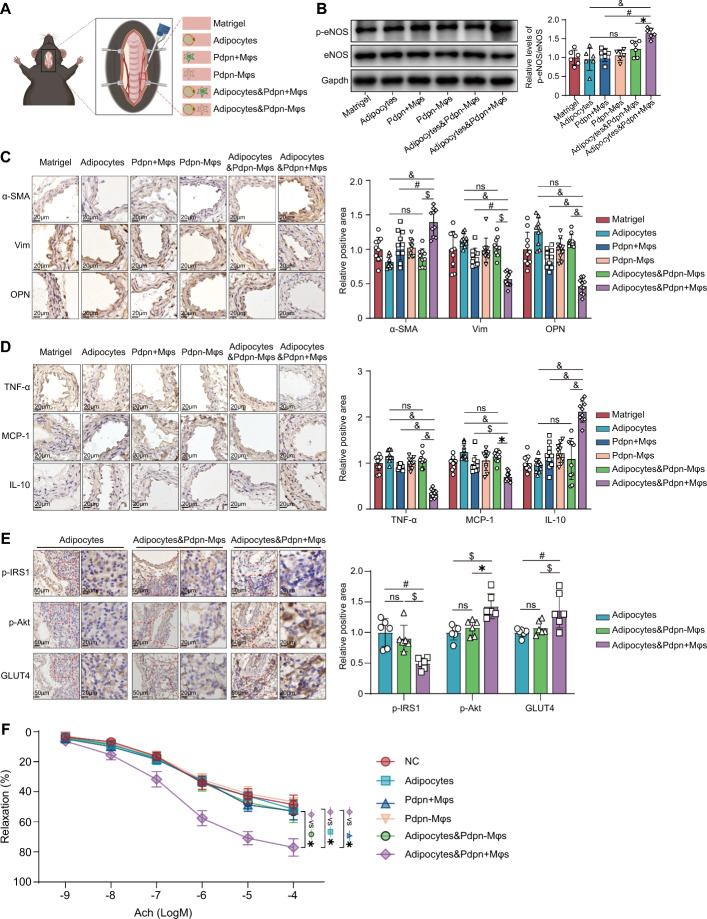
*Pdpn*^+^ macrophages ameliorate vasculopathy in diabetic mice through adipocytes. **A** Schematic representation of cell transplantation in the carotid artery of T2DM mice. Matrigel: matrigel without cells; adipocytes: matrigel containing T2DM-derived adipocytes; *Pdpn *+ Mφs (*Pdpn*-Mφs): matrigel containing *Pdpn*^+^ Mφs (*Pdpn*^−^ Mφs); adipocytes and *Pdpn* + Mφs (adipocytes and *Pdpn*-Mφs): matrigel containing T2DM-derived adipocytes and *Pdpn*^+^ Mφs (*Pdpn*^−^ Mφs). **B** Representative western blot images of phosphorylated eNOS (p-eNOS) and total eNOS (eNOS) in the carotid artery (left panel) and quantitative comparison of p-eNOS/eNOS level of each group (right panel). **C** Representative immunohistochemistry images of α-SMA, vimentin, or OPN staining in the carotid artery of each group (left panel) and their quantitative comparison (right panel). **D** Representative immunohistochemistry images of TNF-α, MCP-1, or IL-10 staining in the carotid artery of each group (left panel) and their quantitative comparison (right panel). **E** Representative immunohistochemistry images of phosphorylated IRS1 (p-IRS1), phosphorylated AKT (p-Akt), or GLUT4 staining in the adipocytes around the carotid artery of each group (left panel) and their quantitative comparison (right panel). **F** Vascular reactivity of isolated carotid arteries from T2DM mice after co-culture with *Pdpn*^+^ Mφs, *Pdpn*^−^ Mφs, or adipocytes. NC: negative control. In **B**–**F**, the grouping is described in **A**. Data are presented as the means ± SDs. One-way ANOVA was used for the statistical analysis for **B**–**E**. Two-way ANOVA followed by Bonferroni post hoc test for **F**. *P < 0.05; $*P* < 0.01; #*P* < 0.001; &*P* < 0.0001; ns: no significance

To further confirm the adipocyte-dependent vasoprotective effects of *Pdpn*^+^ macrophages, we performed in vivo validation experiments using diabetic mice. We sorted *Pdpn*^+^ and *Pdpn*^*−*^ macrophages from mouse PVAT using flow cytometry. Then, we transplanted them and/or primary adipocytes derived from PVAT of T2DM mice around the carotid artery of diabetic mice using Matrigel (Fig. 8A). After 4 weeks, the carotid artery lesions of each group of diabetic mice were examined. The results showed that compared with other group of diabetic mice, including those treated only with *Pdpn*^+^ macrophages, diabetic mice co-transplanted with adipocytes and *Pdpn*^+^ macrophages displayed a significant increase in p-eNOS/eNOS levels, enhanced expression of the contractile VSMC marker α-SMA, and a marked decline in the expression of synthetic VSMC markers vimentin and OPN in the carotid arteries (Fig. 8B, C). Meanwhile, in this group of diabetic mice, the levels of proinflammatory cytokines (TNF-α and MCP-1) were significantly reduced in the carotid arteries, while the level of the anti-inflammatory cytokine IL-10 was markedly increased (Fig. 8D). These results indicated that *Pdpn*^+^ macrophages improved NO signaling dysfunction, inflammation enhancement, and abnormal phenotypic transformation of VSMC in the blood vessels in an adipocyte-dependent manner, thereby ameliorating vascular lesions in diabetic mice. We also found that T2DM-PV-adipocyte treatment exacerbated vascular lesions in T2DM mice, but without statistical significance. Moreover, the transplantation of *Pdpn*^*−*^ macrophages had no significant effects on the aforementioned indicators of vascular injury in T2DM mice (Fig. 8B–D). In addition, immunohistochemical results showed that co-transplantation of adipocytes and *Pdpn*^+^ macrophages significantly reduced IRS phosphorylation (Ser307), increased Akt phosphorylation, and upregulated GLUT4 expression in pericarotid adipocytes (Fig. 8E), indicating that in vivo, *Pdpn*^+^ macrophages could also significantly improve insulin resistance in adipocytes. In vitro co-culture experiments further revealed that the presence of adipocytes is essential for *Pdpn*^+^ macrophages to improve the vascular reactivity and vasodilatory function of the carotid artery. Neither treatment with adipocytes or *Pdpn*^+^ macrophages alone, nor *Pdpn*^*−*^ macrophages co-treatment with adipocytes elicited significant effects (Fig. 8F).

## Discussion

In this study, using scRNA-seq, we revealed the remarkable heterogeneity in the cellular composition and function of the PVAT SVF, as well as its characteristic changes in T2DM. We found significantly enhanced functions related to angiogenesis and proinflammation in all cell populations of the PVAT SVF in T2DM rats. Macrophages were key hubs for cross-talk among PVAT cells. Additionally, we identified a novel *Pdpn*^+^ macrophage subpopulation with vascular protective effects by ameliorating insulin resistance and abnormal adipokine/cytokine expression in adipocytes (Graphical Abstract).

PVAT is adjacent to blood vessels and produces numerous adipokines, cytokines, and vasoactive factors to regulate vascular function [36]. In patients with T2DM, inflammatory cell infiltration and adipocyte enlargement in PVAT cause tissue hypoxia, oxidative stress, and inflammation. These pathological alterations diminish the vascular protective properties of PVAT, fostering abnormal vascular remodeling, and ultimately result in vascular lesions in T2DM [37, 38]. We observed similar increases in inflammation-related functions of various cells in the PVAT SVF of T2DM rats. Moreover, PVAT exerts considerable influences on the proliferation and migration of VSMCs and endothelial cell functions, both of which are critical in initiating the pathological processes of diabetic vascular lesions [39]. Chang et al. found that PVAT played a protective role in atherosclerosis, and vascular functions were significantly impaired in PVAT-deficient mice [40]. However, other studies have demonstrated that expanded PVAT induces dysfunction of vascular endothelial cells (ECs) under pathological conditions such as diabetes [41]. These arguments may be associated with differences in the anatomical location, cellular composition, and function of adipose tissue, as well as the complex crosstalk between cells.

Little is known about the cellular composition and function, as well as intercellular crosstalk in PVAT or their abnormalities in T2DM. Using scRNA-seq, Pan et al. described non-immune cell types in the SVF of murine PVAT, including adipogenic cells, fibroblasts, ECs, and SMCs [42]. Our study systematically elucidated the transcriptome profiles of various cell populations in the SVF of rat PVAT, including immune and non-immune cells, thereby underscoring their heterogeneity and complexity. To fill the knowledge gaps of previous studies, we focused on the composition and function of immune cells in PVAT, particularly macrophages. More importantly, we detailed the abnormalities in cell composition and function of the PVAT SVF in T2DM. These findings contributed to revealing the pathophysiological roles of PVAT and the pathogenesis of T2DM vasculopathy.

Macrophages, which are pivotal in inflammation and vascular dysfunction of PVAT, are generally believed to transition from anti-inflammatory M2 to proinflammatory M1 phenotype as obesity progresses [43, 44]. However, accumulating evidence suggests that M2 macrophages also infiltrate PVAT in obesity, but the mechanisms by which M2 macrophages exacerbate vascular remodeling and dysfunction remain unclear [19, 45]. In addition, macrophages are closely associated with insulin resistance in many cell types. Inflammatory factors secreted by macrophages in adipose tissue, such as TNF-α, are crucial in promoting systemic insulin resistance [46]. In mice, macrophage-specific knockdown of the inflammatory pathway significantly increased insulin sensitivity and protected them from glucose intolerance induced by high-fat diet [47]. However, some studies have shown that blocking inflammatory pathways in macrophages has no effect on insulin sensitivity in obese subjects [48, 49]. Current research primarily focuses on the effect of macrophages in visceral adipose tissue on systemic insulin resistance. However, the influence of macrophages on the insulin sensitivity of other cells in PVAT and its implications for T2DM vasculopathy remain unclear. We identified a macrophage subset with vasoprotective characteristics by ameliorating insulin resistance and abnormal adipokine/cytokine expression in adipocytes. These findings deepened the understanding of the roles of PVAT macrophages in vascular dysfunction.

The advent of scRNA-seq underscores the vast complexity of macrophage phenotypes and functions, surpassing the traditional M1/M2 dichotomy. For example, Cochain et al. found multiple functionally distinct subpopulations of macrophages in atherosclerotic plaques [50]. MacParland et al. identified two types of hepatic macrophages with inflammatory and immunoregulatory functions [51]. Similarly, we discovered four macrophage subpopulations with different functions in PVAT. Among these macrophages, the *Gbp5*^+^ Mφ subpopulation was markedly increased in the PVAT of T2DM rats and potentially played proinflammatory roles in T2DM vasculopathy. Conversely, the proportion of *Pdpn*^+^ Mφs, exhibiting anti-inflammatory and vascular protective effects, was significantly reduced. Recent studies have reported the pivotal role of adipose tissue macrophages (ATMs) in the clearance of dead adipocytes and extracellular lipids. Fatty acid (FA)-activated macrophages contribute to maintaining insulin sensitivity in tissues [52, 53]. In line with this, our findings indicated that highly expressed genes in *Pdpn*^+^ Mφs were involved in FA transport (such as *Fabp4* and *Apoe*) and cholesterol efflux (*Abca1*, *Pltp*, and *Pparg1*). These findings suggested that *Pdpn*^+^ Mφs were involved in lipid metabolism, which might also be related to their roles in alleviating insulin resistance in adipocytes [54].

In our study, PVAT samples were collected from different anatomical locations: the superior thyroid artery in humans, the thoracic aorta in rats, and the carotid artery in mice. Despite these anatomical differences, *Pdpn*^+^ macrophages were significantly reduced in all PVAT samples of T2DM, as confirmed by immunofluorescence. These results suggest that the decrease in *Pdpn*^+^ macrophages is not dependent on the exact PVAT anatomical location. Notably, body weight in T2DM rats was lower than that in control group, while it increased in both T2DM human and mouse models. This discrepancy may reflect distinct mechanisms underlying T2DM development in each model. Studies indicate that approximately 90% of patients with T2DM are overweight or obese, with obesity-induced insulin resistance playing a primary role in T2DM development [55, 56]. In db/db mice, T2DM is induced by *Lepr* gene mutation, often resulting in weight gain [57–59]. In contrast, T2DM in rats is induced by partial pancreatic β-cell destruction with STZ injection and high-fat feeding, leading to weight loss [60, 61]. Despite these differences, we observed a consistent reduction in *Pdpn*^+^ macrophages within PVAT across all T2DM models. These results suggest that the decrease in *Pdpn*^+^ macrophages may be largely independent of body weight changes, but rather associated with insulin resistance and/or deficiency. Therefore, future research should focus on elucidating the causes and mechanisms underlying this reduction of *Pdpn*^+^ macrophages in T2DM.

Pla2g2d, a member of the sPLA2 family, significantly reduced in obese patients and mice, and is negatively correlated with obesity and insulin resistance [62]. In *pla2g2d*^−/−^ mice, inflammatory factor levels in lymph nodes notably increased [63]. Pla2g2d hydrolyzes membrane phospholipids of extracellular vesicles (ECVs) to produce ω3 polyunsaturated fatty acids (ω3 PUFA), such as DHA and EPA [34]. DHA/EPA can bind to *GPR120*, and thereby inhibit proinflammatory cytokine expression in adipocytes, promote adipocyte browning, and improve insulin resistance [64]. In line with these findings, we found that *Pdpn*^+^ macrophages specifically expressed high levels of Pla2g2d, which acted on adipocyte-derived ECVs to produce DHA and EPA. DHA/EPA bound to GPR120 on adipocytes, orchestrating PI3K/Akt pathway activation and NF-κB pathway inhibition. This intricate interaction network ultimately improved insulin resistance and abnormal expression of inflammatory factors in adipocytes of T2DM PVAT. In addition to enhancing the inflammatory response, NF-κB signaling activation also reduces the expression of vasorelaxant factors in adipocytes, leading to vascular dysfunction. Through the Pla2g2d-DHA/EPA-GPR120-NFκB/Akt axis, *Pdpn*^+^ macrophages exerted a protective effect on diabetic vascular lesions. Furthermore, our in vivo cell transplantation experiment confirmed that *Pdpn*^+^ macrophages ameliorated vascular lesions in T2DM mice by modulating adipocyte function. These findings suggested that *Pdpn*^+^ Mφs could be a potential therapeutic target for vascular complications of T2DM, warranting further in-depth investigation.

The number of NK cells has been reported to significantly increase in epididymal adipose tissue in mice fed a high-fat diet [65]. NK cells in visceral adipose tissue are implicated in obesity-induced insulin resistance and adipose tissue inflammation through IFN-γ release [66]. In our study, we found an increase in NK cell numbers in the PVAT of T2DM rats. Two T2DM-specific NK cell subsets, *Klra22*^+^ and *S100a4*^+^ NK cells, were identified in PVAT. The *Klra22*^+^ NK cell subset was mainly involved in IFN-γ-mediated inflammatory pathways, possibly contributing to chronic inflammation in adipose tissue. The energy metabolism of the *S100a4*^+^ NK cell subset was significantly enhanced, potentially providing more biosynthetic precursors for granzyme and perforin production [67, 68]. Moreover, the enhanced glycolysis and OXPHOS significantly increased IFN-γ production [69], promoting macrophage recruitment and infiltration, thereby establishing a proinflammatory positive-feedback loop. However, the precise roles and mechanisms of NK cells of PVAT in T2DM vascular lesions remain poorly understood, and experimental validation of our findings is still warranted.

It should be noted that there is an important limitation in this study, namely the use of two pooled samples for scRNA-seq analysis (9–10 rats per group). Although sample pooling minimizes the impact of inter-individual variability on the results, it overlooks the biological heterogeneity among individuals. To address this limitation, we validated our primary finding from the scRNA-seq analysis—the decrease in *Pdpn*^+^ macrophages—using immunofluorescence co-localization experiments across three species (rats, mice, and humans; *n* > 5 per group). This approach allowed us to demonstrate the variability within the group, namely how individual subjects in each group differ from each other, something we are blind to in the scRNA-seq analysis.

## Conclusions

In conclusion, our study unveiled the single-cell landscape of the SVF of PVAT and identified its abnormalities in T2DM. Moreover, we discovered a novel *Pdpn*^+^ macrophage subset that ameliorated insulin resistance in adipocytes and exerted anti-inflammatory and vascular protective effects through the Pla2g2d-DHA/EPA-GPR120 axis. Our findings enhanced the understanding of the cell composition and function of PVAT, the roles of PVAT macrophages in vascular function and pathology, and immune–metabolic cross-talk. However, it is noteworthy that although we demonstrated the beneficial effects of *Pdpn*^+^ macrophages on vascular function through in vitro and in vivo experiments, their therapeutic potential still requires further validation.

## Supplementary Information

Additional file 1: Figure S1. Pathophysiological features of HFD/STZ-induced diabetic rats. Figure S2. Heatmap of the relative expression levels of representative genes from the top 10 marker genes in each cell population of the SVF of rat PVAT. Figure S3. Subpopulations and functional enrichment analysis of ADSCs, SMCs and NKs in the SVF of rat PVAT. Figure S4. The results of WGCNA and functional changes of immune cells in the SVF of PVAT in T2DM rats. Figure S5. Abnormal communications among various cell populations of PVAT SVF in T2DM rats using CellChat and Celltalker. Figure S6. The proportions of *Pdpn*^+^ macrophages, pathophysiological features of *db/db* mice, selective expression of Pla2g2d in *Pdpn*^+^ macrophages, and the efficiency of siRNAs targeted to Pla2g2d or GPR120. Table S1. Physiological characteristics of rats, mice, and clinical data of human subjects. Table S2. Antibodies used in the present study. Table S3. Sequences of GPR120 or Pla2g2d siRNA used in the present study. Table S4. Primer sequences used for qRT-PCR analysis in the present study.

Additional file 2. Original western blots.

## Data Availability

The data supporting this study are available from the corresponding authors upon reasonable request. The scRNA-seq data have been archived in the NCBI Sequence Read Archive database (accession number SRX25842308, SRX25842309). The processed gene expression matrices are deposited in the NCBI Gene Expression Omnibus repository (accession number GSE275779). All code used in this study is publicly accessible at https://doi.org/10.5281/zenodo.13651502.

## Abbreviations

PVAT: Perivascular adipose tissue
SVF: Stromal vascular fraction
T2DM: Type 2 diabetes mellitus
NO: Nitric oxide
scRNA-seq: Single-cell RNA sequencing
ADSCs: Adipose-derived stem cells
SMCs: Smooth muscle cells
ECs: Endothelial cells
Macs: Macrophages
DCs: Dendritic cells
VEGF: Vascular endothelial growth factor
WGCNA: Weighted correlation network analysis
VSMC: Vascular smooth muscle cell
ROS: Reactive oxygen species
Pla2g2d: Phospholipase A2 IIG IID
ω3 PUFA: ω3 Polyunsaturated fatty acids
