# Supplementary material for: Single-cell view and a novel protective macrophage subset in perivascular adipose tissue in T2DM
Source: Cell Mol Biol Lett. 2024 Dec 3;29:148. doi: 10.1186/s11658-024-00668-5 (PMC11616190; doi:10.1186/s11658-024-00668-5)
Supplement: Supplementary file 1 — Additional file 1: Figure S1. Pathophysiological features of HFD/STZ-induced diabetic rats. Figure S2. Heatmap of the relative expression levels of representative genes from the top 10 marker genes in each cell population of the SVF of rat PVAT. Figure S3. Subpopulations and functional enrichment analysis of ADSCs, SMCs and NKs in the SVF of rat PVAT. Figure S4. The results of WGCNA and functional changes of immune cells in the SVF of PVAT in T2DM rats. Figure S5. Abnormal communications among various cell populations of PVAT SVF in T2DM rats using CellChat and Celltalker. Figure S6. The proportions of Pdpn+ macrophages, pathophysiological features of db/db mice, selective expression of Pla2g2d in Pdpn+ macrophages, and the efficiency of siRNAs targeted to Pla2g2d or GPR120. Table S1. Physiological characteristics of rats, mice, and clinical data of human subjects. Table S2. Antibodies used in the present study. Table S3. Sequences of GPR120 or Pla2g2d siRNA used in the present study. Table S4. Primer sequences used for qRT-PCR analysis in the present study. [file 11658_2024_668_MOESM1_ESM.docx]

**Additional Figures and Tables**

Single-cell view and a novel protective macrophage subset in perivascular adipose tissue in T2DM

Jiaxuan Li^1,2,3,4^†, Zhenyu Tian^1^†, Tongxue Zhang^1^, Jiajia Jin^1^, Xinjie Zhang^5^, Panpan Xie^6^, Haiyan Lin^2^, Junfei Gu^1,2^, Yingjie Wu^3,4^, Xiaowei Wang^1^, Shucui Zhang^1^, Xuefang Yan^1^, Dong Guo^7^*, Zhe Wang^2^* and Qunye Zhang^1,7^*

^1^State Key Laboratory for Innovation and Transformation of Luobing Theory; Key Laboratory of Cardiovascular Remodeling and Function Research, Chinese Ministry of Education, Chinese National Health Commission and Chinese Academy of Medical Sciences; Department of Cardiology, Qilu Hospital of Shandong University, Jinan, 250012, China.

^2^Department of Endocrinology; Department of Geriatrics, Shandong Provincial Hospital Affiliated to Shandong First Medical University, Jinan, 250021, China.

^3^Shandong Provincial Hospital, Shandong Laboratory Animal Center, Science and Technology Innovation Center, Shandong First Medical University & Shandong Academy of Medical Science, Jinan, 250021, China.

^4^Key Laboratory of Endocrine Glucose & Lipids Metabolism and Brain Aging, Chinese Ministry of Education, Shandong First Medical University, Jinan, 250021, China.

^5^Department of Biology, University College London, London NW1 2HE, UK.

^6^Department of Breast and Thyroid Surgery, Liaocheng People's Hospital, Liaocheng, 252000, China.

^7^Department of Neurology, Liaocheng People's Hospital, Liaocheng, 252000, China.

†These authors contributed equally: Jiaxuan Li, Zhenyu Tian.

*Corresponding Author:

Dong Guo, E-mail: guodonglc@126.com. Telephone: +86-0[635-8272138](https://www.google.com.hk/search?q=%E8%81%8A%E5%9F%8E%E5%B8%82%E4%BA%BA%E6%B0%91%E5%8C%BB%E9%99%A2+%E7%94%B5%E8%AF%9D&sca_esv=ba9c76241f1fe624&sca_upv=1&source=hp&ei=T0TJZob5DtfX1e8P_J7o2QM&iflsig=AL9hbdgAAAAAZslSX4gWaHIL9jxPfZQJe2Kuy50khO1W&ved=0ahUKEwjG3Mi0yYyIAxXXa_UHHXwPOjsQ4dUDCA0&uact=5&oq=%E8%81%8A%E5%9F%8E%E5%B8%82%E4%BA%BA%E6%B0%91%E5%8C%BB%E9%99%A2+%E7%94%B5%E8%AF%9D&gs_lp=Egdnd3Mtd2l6IhzogYrln47luILkurrmsJHljLvpmaIg55S16K-dMgUQIRigAUiIOVCwBViEN3ABeACQAQGYAecCoAGqMKoBCDAuMzIuMi4xuAEDyAEA-AEBmAIZoALkIagCAMICCxAuGIAEGNEDGMcBwgIFEAAYgATCAgUQLhiABMICCxAuGIAEGMcBGK8BwgINEC4YgAQYxwEYDRivAcICBBAAGB7CAgQQLhgewgIGEAAYDRgewgIGEC4YDRgewgIKEC4YgAQY1AIYDcICBxAAGIAEGA3CAgcQLhiABBgNwgIGEAAYHhgPwgIIEAAYgAQYogTCAgkQIRigARgKGCrCAgcQIRigARgKmAMBkgcGMC4yNC4xoAelsQE&sclient=gws-wiz).

Zhe Wang, E-mail: wangzhe.zqy@email.sdu.edu.cn. Telephone: +86-0531-68776130.

Qunye Zhang, E-mail: wz.zhangqy@sdu.edu.cn. Telephone: +86-0531-82169256.

**This file includes:**

**Supplementary Figures**

**Figure S1.** Pathophysiological features of HFD/STZ-induced diabetic rats.

**Figure S2.** Heatmap of the relative expression levels of representative genes from the top 10 marker genes in each cell population of the SVF of rat PVAT.

**Figure S3.** Subpopulations and functional enrichment analysis of ADSCs, SMCs and NKs in the SVF of rat PVAT.

**Figure S4.** The results of WGCNA and functional enrichment analysis of immune cells in the SVF of PVAT in T2DM rats.

**Figure S5.** Abnormal communications among various cell populations of PVAT SVF in T2DM rats usinag CellChat and Celltalker.

**Figure S6.** The proportions of *Pdpn*^+^ macrophages, pathophysiological features of *db/db* mice, selective expression of Pla2g2d in *Pdpn*^+^ macrophages, and the efficiency of siRNAs targeted to Pla2g2d or GPR120.

**Supplementary Tables**

**Table S1.** Physiological characteristics of rats, mice, and clinical data of human subjects.

**Table S2.** Antibodies used in the present study.

**Table S3.** Sequences of GPR120 or Pla2g2d siRNA used in the present study.

**Table S4.** Primer sequences used for qRT-PCR analysis in the present study.

**Supplementary Figures**

**Figure S1**

**
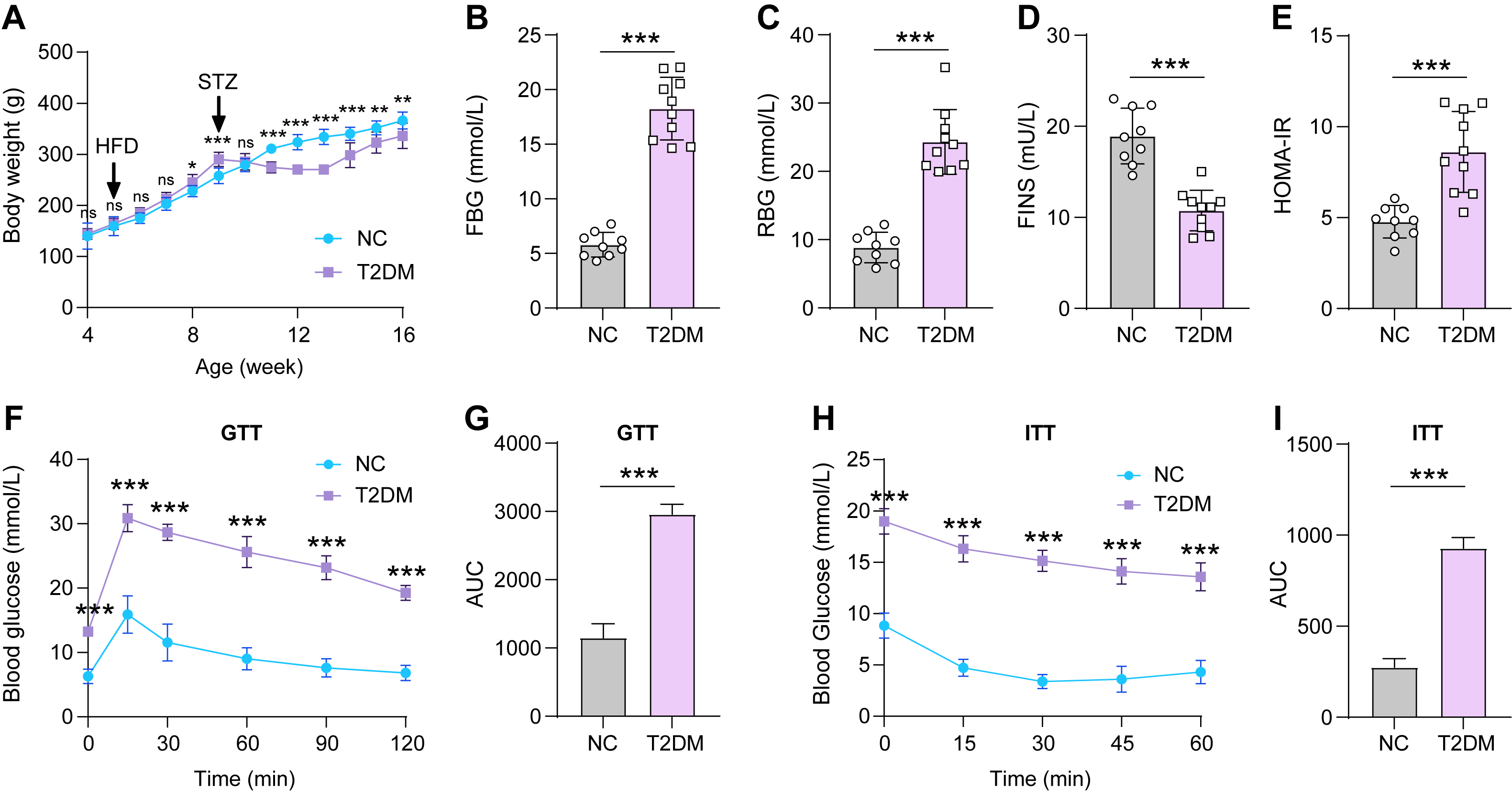
**

**Figure S1. Pathophysiological features of HFD/STZ-induced diabetic rats. (A)** Body weight over time for normal (NC) and T2DM (T2DM) rats groups. **(B-E)** Comparison of fasting blood glucose (FBG) **(B)**, random blood glucose (RBG) **(C)**, fasting insulin (FINS) **(D)**, and homeostasis model assessment for insulin resistance (HOMA-IR) **(E)** levels between NC and T2DM groups. **(F, G)** The results of glucose tolerance test (GTT) **(F)** and area under the curve (AUC) **(G)** between NC and T2DM groups. **(H, I)** The results of insulin tolerance test (ITT) **(H)** and AUC **(I)** between NC and T2DM groups. Data are presented as mean ± SD. Two-way ANOVA with Bonferroni post-hoc analysis was used for **A** and **F**, and two tailed Student's t-test was used for **B-E, G** and **I**. *: P<0.05; **: P<0.01; ***: P<0.001.

**Figure S2**


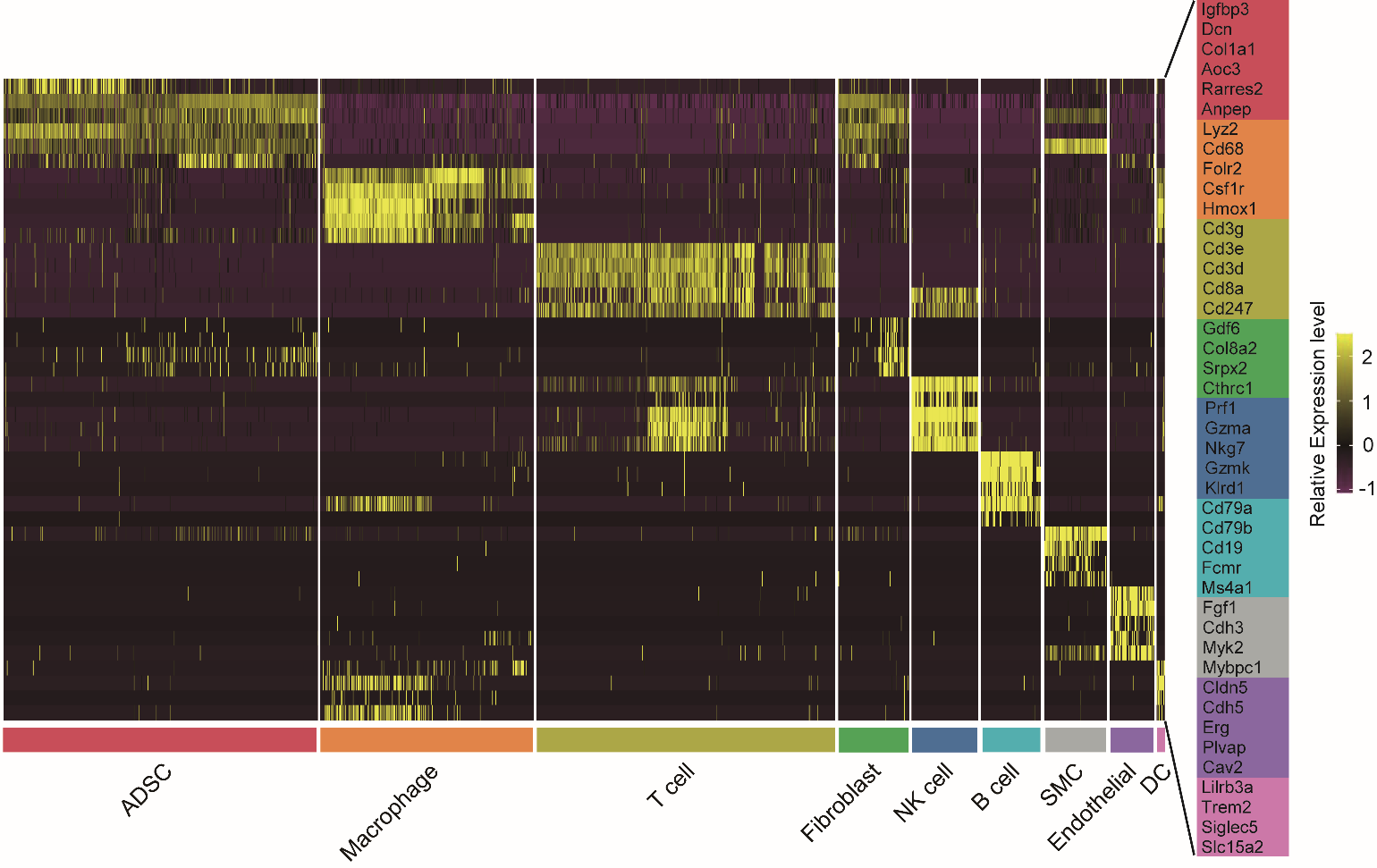


**Figure S2. Heatmap of the relative expression levels of representative genes from the top 10 marker genes in each cell population of the SVF of rat PVAT.** Relative expression level: the expression of genes in each population compared to all other cell populations in the SVF of rat PVAT. Mann-Whitney U test with Bonferroni correction was used for the statistical analysis.

**Figure S3**


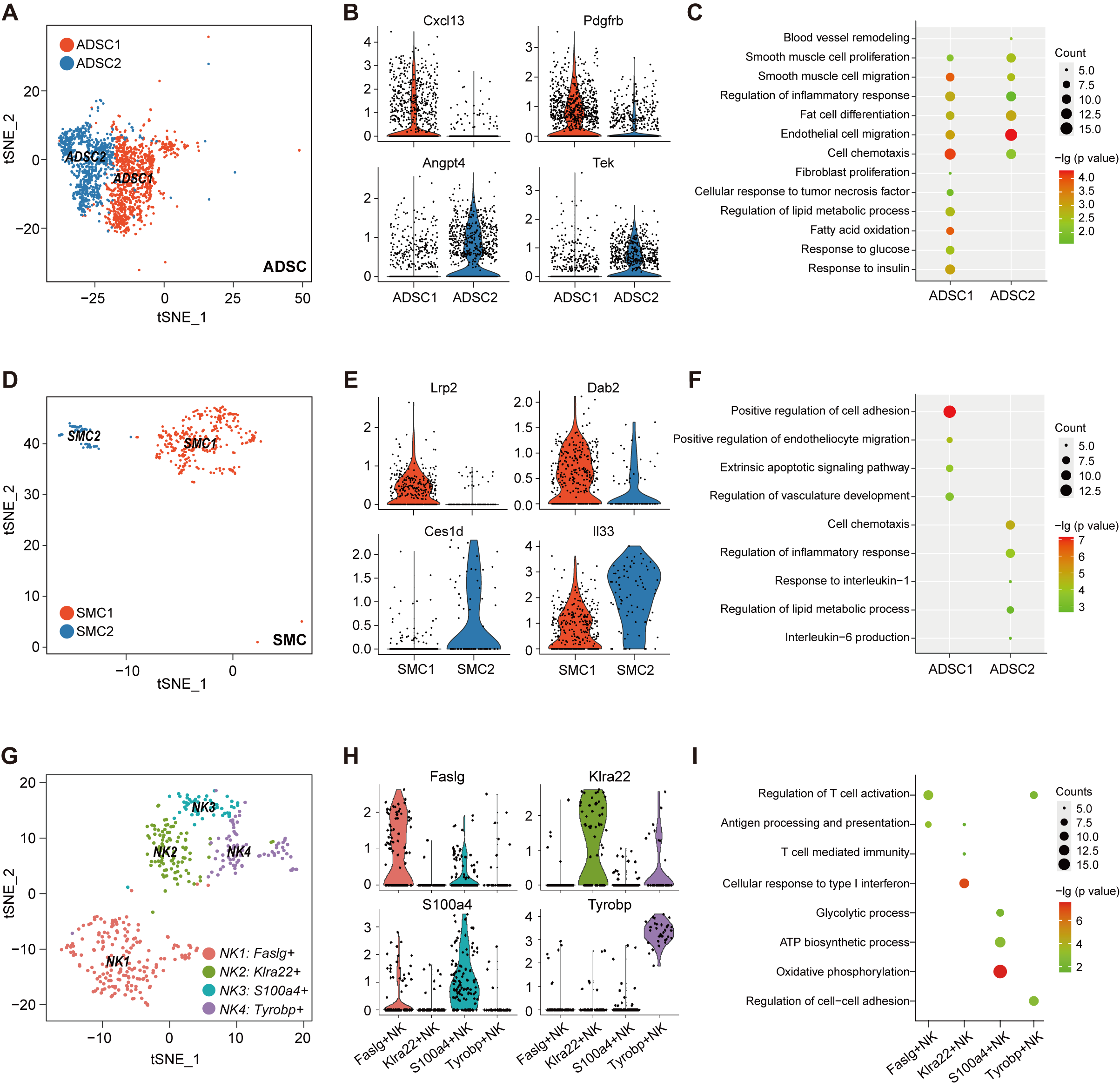


**Figure S3. Subpopulations and functional enrichment analysis of ADSCs, SMCs and NKs in the SVF of rat PVAT. (A)** The t-SNE plot depicting ADSC subpopulations in the SVF of PVAT in rats. **(B)** Violin plots of the normalized expression levels of marker genes in each ADSC subpopulation. **(C)** Functional enrichment analysis of each ADSC subpopulation in the SVF of rat PVAT. **(D)** The t-SNE plot showing SMC subpopulations in the SVF of PVAT in rats. **(E)** Violin plots of the normalized expression levels of marker genes in each SMC subpopulation. **(F)** Functional enrichment analysis of each SMC subpopulation in the SVF of rat PVAT. **(G)** The t-SNE plot showing NK cell subpopulations in the SVF of PVAT. **(H)** Violin plots showing the expression levels of marker genes in each NK cell subpopulation of the SVF of PVAT. **(I)** Functional enrichment analysis of each NK cell subpopulation in the SVF of PVAT. In **C**, **F, and I**, bubble size and color represent the number of genes involved in the corresponding biological process and the significance of enrichment analysis, respectively. Fisher's exact test with Benjamini-Hochberg FDR multiple-test correction was used for the statistical analysis in **C, F, and I**.

**Figure S4**

**
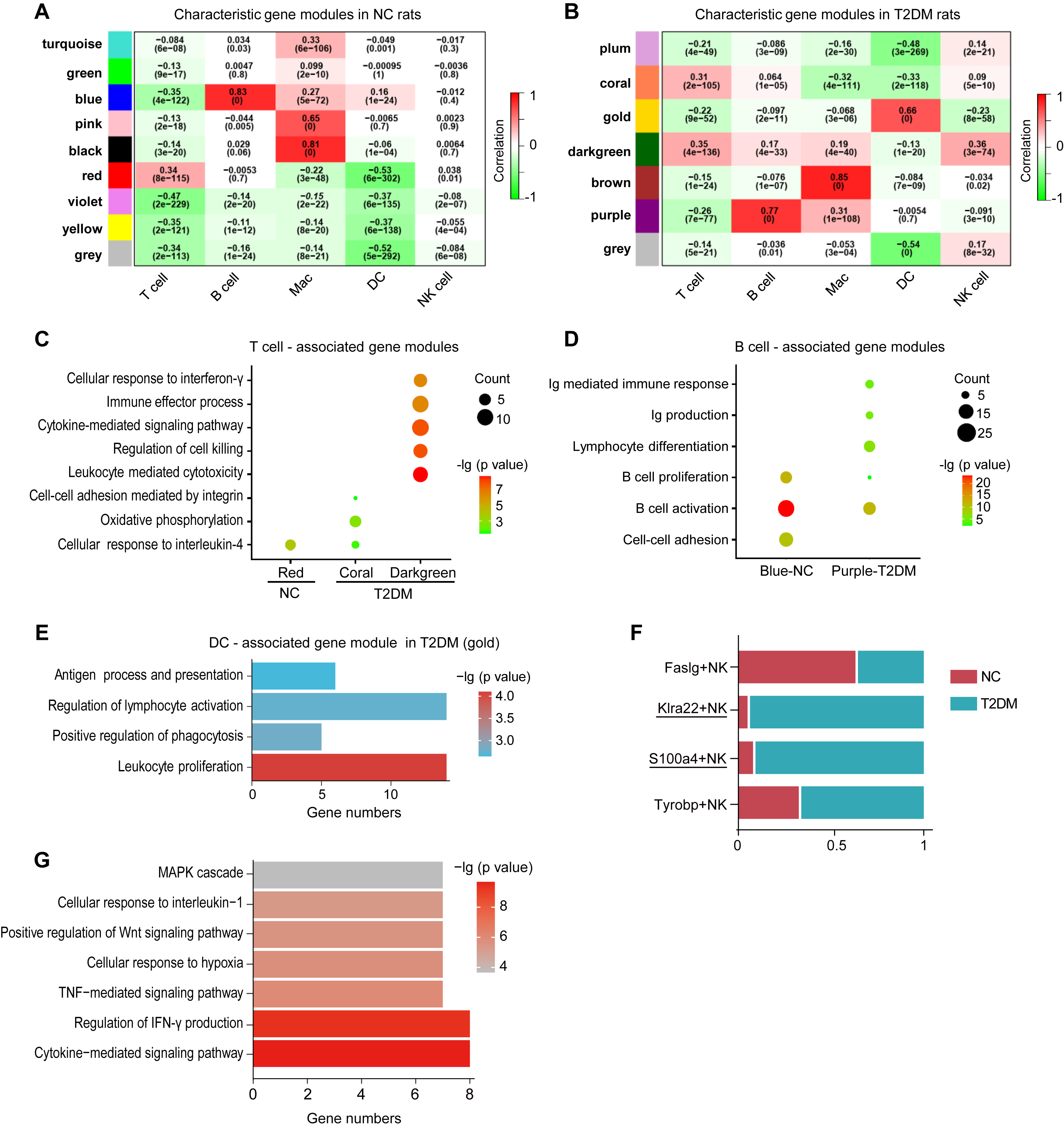
**

**Figure S4. The results of WGCNA and functional enrichment analysis of immune cells in the SVF of PVAT in T2DM rats. (A, B)** Characteristic (significantly related) gene modules of cell populations in the SVF of PVAT in normal (**A**, NC) and type 2 diabetes (**B**, T2DM) rats revealed by weighted correlation network analysis (WGCNA). In each rectangle, the upper number indicates the correlation coefficient, and the lower number in brackets indicates the corrected p value. **(C, D)** Functional enrichment analysis of genes in each characteristic module of T cell **(C)** or B cell **(D)** of the SVF of PVAT in NC and T2DM rats. Bubble size and color represent the number of genes involved in the corresponding biological process and the significance of enrichment analysis, respectively. **(E)** Functional enrichment analysis of genes in the DC-associated module in T2DM rats. Color bar represents the significance of enrichment analysis. **(F)** Bar plots illustrating the proportion of the number of indicated NK cell subpopulation in the SVF of PVAT data of normal (red) or T2DM (blue) rats, compared to the total number of the corresponding cell subpopulation in the integrated data of the SVF of PVAT. **(G)** Functional enrichment analysis of the genes significantly upregulated in NK cells in the PVAT SVF of T2DM rats. The color bar represents the significance of enrichment analysis. Fisher's exact test with Benjamini-Hochberg FDR multiple-test correction was used for the statistical analysis in **C**, **D**, **E, and G**. Chi-square test of independence was used for the statistical analysis in **F**.

**Figure S5**


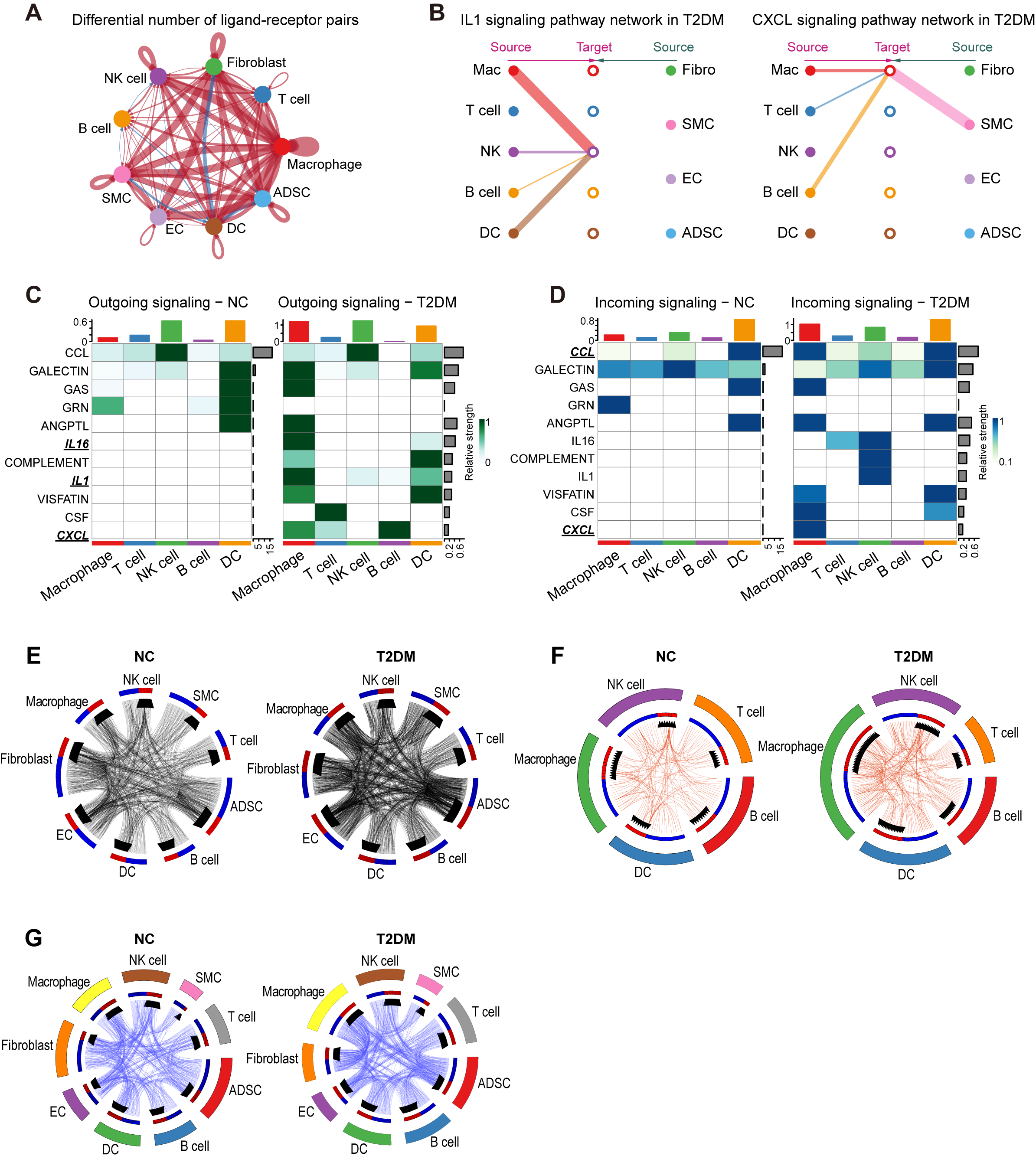


**Figure S5. Abnormal communications among various cell populations of PVAT SVF in T2DM rats using CellChat and Celltalker. (A)** Circle plot showing the differential numbers of ligand-receptor pairs in the SVF of PVAT in T2DM rats compared with those in normal rats. Edge width: the differential numbers of ligand-receptor pairs. Red (Blue): increase (reduction). **(B)** Hierarchical plots of inferred intercellular communication of IL1 (left) and CXCL (right) signaling pathway in PVAT SVF of T2DM rats. Circle color: cell types. Edge width: the communication probability. Edge colors are consistent with the cells of signaling source. Communication probability: the average expression of all ligands and receptors related to a certain signaling pathway in each cell population. **(C, D)** Heatmap of the interaction strength of outgoing **(C)** and incoming **(D)** signaling pathways in various immune cells of the SVF of PVAT in normal (NC) and T2DM rats. Color bar: the relative interaction strength of a signaling pathway in different cell populations. The top bar shows the sum of the relative interaction strength of all signaling pathways in a cell population. The right bar indicates the sum of the relative interaction strength of a signaling pathway in all cell populations. **(E)** Comparative cell-cell communication networks between various cell populations in the SVF of PVAT. Cells emitting arrows express ligands, while cells receiving arrows express receptors. **(F, G)** Cell-cell communication between immune cells **(F)** or between immune cells and non-immune cells **(G)** in the SVF of PVAT. Analysis of cell communication was performed using CellChat for **A-D**, and Celltalker for **E-G**.

**Figure S6**


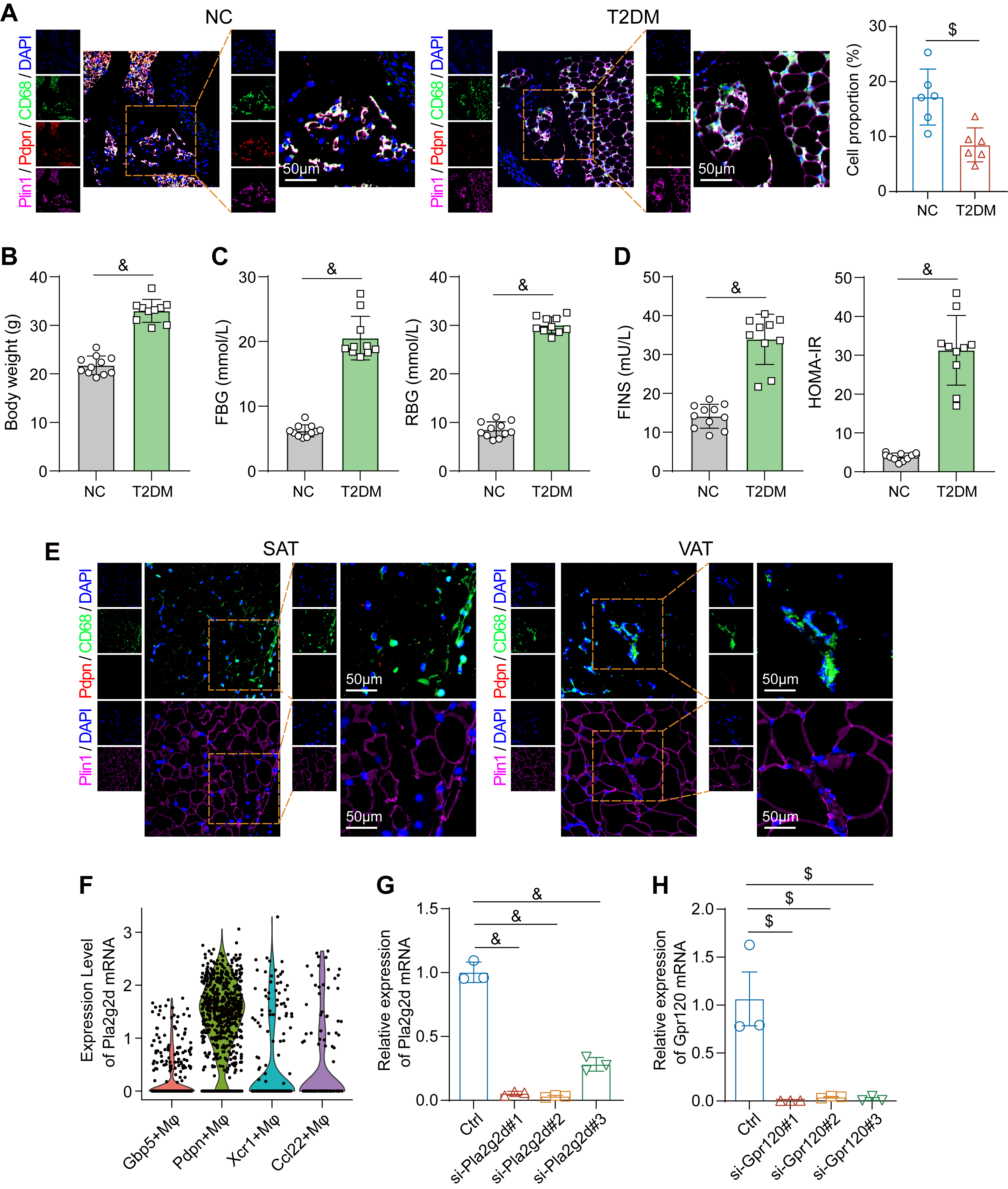


**Figure S6. The proportions of *Pdpn*^+^ macrophages, pathophysiological features of *db/db* mice, selective expression of Pla2g2d in *Pdpn*^+^ macrophages, and the efficiency of siRNAs targeted to Pla2g2d or GPR120. (A)** Left panel: representative immunofluorescence images of *Cd68*^+^*Pdpn*^+^ Mφs in the PVAT of mice. CD68: green; Pdpn: red; the adipocyte marker perilipin 1 (Plin1): pink. Right panel: comparison of the proportions of *Cd68*^+^*Pdpn*^+^ Mφs to *Cd68*^+^ Mφs in PVAT of normal (NC) and T2DM (T2DM) mice. **(B-D)** Comparison of body weight (BW) **(B)**, fasting blood glucose (FBG) , random blood glucose (RBG) **(C)**, fasting insulin (FINS), homeostasis model assessment for insulin resistance (HOMA-IR) **(D)** levels between NC and T2DM groups. **(E)** Representative immunofluorescence images of *Cd68*^+^*Pdpn*^+^ Mφs in subcutaneous adipose tissue (SAT) and visceral adipose tissue (VAT) in rats. CD68 (green), Pdpn (red), and the adipocyte marker perilipin 1 (Plin1, pink) were stained on two consecutive sections: the first section (top) was stained for CD68 (green) and Pdpn (red), and the second section (bottom) was stained for perilipin 1 (Plin1, pink). **(F)** Violin plot of the normalized expression levels of Pla2g2d mRNA in each macrophage subpopulation of PVAT SVF in rats. **(G)** Efficiency of si-Pla2g2d for suppressing Pla2g2d expression in *Pdpn*^+^ macrophages confirmed by qPCR. **(H)** Efficiency of si-GPR120 for inhibiting GPR120 expression in primary adipocytes isolated from PVAT confirmed by qPCR. Data are presented as means ± SDs. The two-tailed Student’s t test was used for the statistical analysis in **A-D**. One-way ANOVA was used for the statistical analysis in **G** and **H**. $: P < 0.01; &: P < 0.0001.

**Supplementary Tables**

**Table S1. Physiological characteristics of rats, mice, and clinical data of human subjects**

| **Human clinical data** | **Healthy (n = 6)** | **T2DM (n = 6)** | **P value** |
| --- | --- | --- | --- |
| Sex (male/female) | 4/2 | 4/2 | 1 |
| Age (year) | 48.5 ± 9 | 51.5 ± 7.3 | 0.541 |
| BMI (kg/m2) | 23.1 ± 2.08 | 29.36 ± 2.40 | < 0.001*** |
| FBG (mmol/L) | 4.65 ± 0.29 | 10.02 ± 2.56 | < 0.001*** |
| OGTT 2hrs (mM) | 6.53 ± 0.75 | 16.74 ± 4.74 | < 0.001*** |
| FINS (mU/L) | 11.7 ± 2.70 | 21.12 ± 6.11 | 0.004** |
| HOMA-IR | 2.41 ± 0.54 | 9.75 ± 4.40 | 0.002** |
| GHbA1c (%) | 5.72 ± 0.25 | 10.00 ± 2.55 | 0.002** |
| **Rat physiological characteristics** | **Control (n = 9)** | **T2DM (n = 10)** | **P value** |
| Type | Wistar | Wistar | - |
| Genotype | WT | WT | - |
| Age (week) | 16 | 16 | - |
| Sex (male/female) | male | male | - |
| BW (g) | 366.03 ± 16.48 | 336.32 ± 24.75 | 0.007** |
| FBG (mmol/L) | 5.80 ± 1.12 | 18.41 ± 2.87 | < 0.001*** |
| RBG (mmol/L) | 8.84 ± 2.24 | 24.39 ± 4.74 | < 0.001*** |
| FINS (mU/L) | 18.72 ± 3.07 | 10.53 ± 2.23 | < 0.001*** |
| HOMA-IR | 4.77 ± 0.89 | 8.62 ± 2.23 | < 0.001*** |
| GTT 2hrs (mM) | 6.83 ± 1.18 | 19.27 ± 1.56 | < 0.001*** |
| ITT 2hrs (mM) | 4.31 ± 1.14 | 14.58 ± 1.37 | < 0.001*** |
| **Mice physiological characteristics** | **WT (n = 11)** | **db/db (n = 10)** | **P value** |
| Strain | C57BLKS/J | C57BLKS/J | - |
| Genotype | WT Cat.no: #000662 | BKS.Cg-Dock7m +/+ Leprdb/J Cat.no: #000642 | - |
| Age (week) | 6 | 6 | - |
| Sex (male/female) | male | male | - |
| BW (g) | 21.74 ± 1.93 | 32.97 ± 2.36 | < 0.001*** |
| FBG (mmol/L) | 6.20 ± 0.92 | 20.53 ± 3.36 | < 0.001*** |
| RBG (mmol/L) | 8.51 ± 1.59 | 30.05 ± 1.68 | < 0.001*** |
| FINS (mU/L) | 14.08 ± 3.07 | 33.90 ± 6.49 | < 0.001*** |
| HOMA-IR | 3.88 ± 0.94 | 31.27 ± 8.97 | < 0.001*** |

Note: Data are shown as mean ± SD. P-values were determined using the chi-square test (sex) or two tailed Student's t-test (age, BW, BMI, FBG, RBG, OGTT 2hrs, GTT 2hrs, ITT 2hrs, FINS, HOMA-IR, GHbA1c). BW, body weight; BMI, body mass index; FBG, fasting blood glucose; RBG, random blood glucose; OGTT 2hrs, oral glucose tolerance test; FINS, fasting insulin; HOMA-IR, homeostasis model assessment for insulin resistance, calculated as FBG (mmol/L) × FINS (mU/L)/22.5; GHbA1c, Glycated haemoglobin; GTT 2hrs, glucose tolerance test; ITT 2hrs, insulin tolerance test. *: P < 0.05; **: P < 0.01; ***: P < 0.001.

**Table S2. Antibodies used in the present study**

| **Antibodies** | **Source** | **Cat. No.** | **Dilution** |
| --- | --- | --- | --- |
| Anti-GAPDH antibody | Cell Signaling | 2118S | 1:5000 (WB) |
| Anti-β-Actin antibody | Proteintech | 66009-1-Ig | 1:5000 (WB) |
| Anti-Akt antibody | Proteintech | 60203-2-Ig | 1:5000 (WB) |
| Anti-Akt (phospho Ser473) antibody | Proteintech | 66444-1-Ig | 1:5000 (WB) / 1:200 (IHC) |
| Anti-eNOS antibody | Proteintech | 27120-1-AP | 1:1000 (WB) |
| Anti-eNOS (phospho Ser1177) antibody | Abcam | Ab215717 | 1:500 (WB) |
| Anti-eNOS (phospho Ser1177) antibody | Abclonal | AP0515 | 1:1000 (WB) |
| Anti-GLUT4 antibody | Proteintech | 66846-1-Ig | 1:200 (IHC) |
| Anti-IRS1 antibody | Proteintech | 17509-1-AP | 1:1000 (WB) |
| Anti-IRS1 (phospho Ser307) antibody | Abcam | Ab5599 | 1:1000 (WB) / 1:100 (IHC) |
| Anti-NF-κB antibody | Cell Signaling | 8242 | 1:1000 (WB) |
| Anti-NF-κB (phospho Ser 536) antibody | Cell Signaling | 3031 | 1:1000 (WB) |
| Anti-PI3K antibody | Proteintech | 60225-1-Ig | 1:1000 (WB) |
| Anti-Perilipin1 antibody | Abcam | Ab3526 | 1:200 (IF) |
| Anti-Cd68 antibody | Genetex | GTX41865 | 1:200 (IF) |
| Anti-Pdpn antibody | Santa Cruz | Sc-166906 | 1:200 (IF) |
| Anti-Cd68 antibody | Santa Cruz | Sc-20060 | 1:20 (FC) |
| Anti-Pdpn antibody | Boster | A01124-2 | 1:100 (FC) |
| Anti-α-SMA antibody | Proteintech | 14395-1-AP | 1:1000 (IHC) |
| Anti-Vimentin antibody | Proteintech | 10366-1-AP | 1:1000 (IHC) |
| Anti-OPN antibody | Proteintech | 22952-1-AP | 1:50 (IHC) |
| Anti-TNF-α antibody | Proteintech | 60291-1-Ig | 1:100 (IHC) |
| Anti-MCP-1 antibody | Proteintech | 66272-1-Ig | 1:100 (IHC) |
| Anti-IL-10 antibody | Proteintech | 60269-1-Ig | 1:200 (IHC) |
| Anti-Pla2g2d antibody | Novus Biologicals | NBP2-94062 | 1:1000 (WB) |
| Anti-Cd68 antibody (APC-conjugated) | BioLegend | 137007 | 1:100 (FC) |
| Anti-Pdpn antibody (PE-conjugated) | BioLegend | 127407 | 1:100 (FC) |
| Goat anti-mouse IgG | Proteintech | SA00001-1 | 1:5000 (WB) |
| Goat anti-rabbit IgG | Proteintech | SA00001-2 | 1:5000 (WB) |
| Goat anti-mouse IgG (488-conjugated) | Proteintech | SA00013-1 | 1:200 (IF) |
| Goat anti-rabbit IgG (488-conjugated) | Proteintech | SA00013-2 | 1:200 (IF) |
| Goat anti-mouse IgG (594-conjugated) | Proteintech | SA00013-3 | 1:200 (IF) |
| Goat anti-rabbit IgG (594-conjugated) | Proteintech | SA00013-4 | 1:200 (IF) |
| Goat anti-rabbit IgG (Cy5-conjugated) | Abcam | Ab6564 | 1:500 (IF) |
| Goat anti-rat IgG (488-conjugated) | Abcam | Ab150157 | 1:500 (IF) |
| Goat anti-rabbit IgG (PE-conjugated) | Proteintech | SA00008-2 | 1:100 (FC) |
| Rat anti-mouse IgG (APC-conjugated) | BioLegend | 406609 | 1:100 (FC) |

Note: WB: Western Blot; IHC: Immunohistochemistry; IF: Immunofluorescence, FC: Flow Cytometry

**Table S3. Sequences of GPR120 or Pla2g2d siRNA used in the present study**

| **Genes** | **siRNA sequences** |
| --- | --- |
| *siGPR120#1* | Forward 5’-CCCAUACUGUACAACAUGUTT-3’ |
|  | Reverse 5’-ACAUGUUGUACAGUAUGGGTT-3’ |
| *siGPR120#2* | Forward 5’-GGACUGGUCAUUGUGAUCATT-3’ |
|  | Reverse 5’-UGAUCACAAUGACCAGUCCTT-3’ |
| *siGPR120#3* | Forward 5’-GCUCAUCUUUGUCGUCUCATT-3’ |
|  | Reverse 5’-UGAGACGACAAAGAUGAGCTT-3’ |
| *SiPLA2G2D#1* | Forward 5’-GCAAGAGCCUGACAGACAACU-3’ |
|  | Reverse 5’-UUGUCUGUCAGGCUCUUGCAU-3’ |
| *siPLA2G2D#2* | Forward 5’-GAAGCAUGAUUGUUGCUAUGC-3’ |
|  | Reverse 5’-AUAGCAACAAUCAUGCUUCUG-3’ |
| *siPLA2G2D#3* | Forward 5’-GCAAGACUCCAGCAUGCUAA-3’ |
|  | Reverse 5’-UAGCAUGCUGGAGUCUUGCCU-3’ |
| *Ctrl (si-NC)* | Forward 5’-UUCUCCGAACGUGUCACGUTT-3’ |
|  | Reverse 5’-ACGUGACACGUUCGGAGAATT-3’ |

**Table S4. Primer sequences used for qRT-PCR analysis in the present study**

| **Genes** | **Primer sequences** |
| --- | --- |
| *TNF-α (Mus)* | Forward 5’- ATGTCTCAGCCTCTTCTCATTC-3’ |
|  | Reverse 5’- GCTTGTCACTCGAATTTTGAGA-3’ |
| *IL-6 (Mus)* | Forward 5’- CTCCCAACAGACCTGTCTATAC-3’ |
|  | Reverse 5’- CCATTGCACAACTCTTTTCTCA-3’ |
| *MCP-1 (Mus)* | Forward 5’- ATGTCTCAGCCTCTTCTCATTC-3’ |
|  | Reverse 5’- GCTTGTCACTCGAATTTTGAGA-3’ |
| *Adiponectin (Mus)* | Forward 5’- CCAATGTACCCATTCGCTTTAC-3’ |
|  | Reverse 5’- GAAGTAGTAGAGTCCCGGAATG-3’ |
| *Omentin (Mus)* | Forward 5’- TGTGCTTCTCTGCCATTCGGTATTC-3’ |
|  | Reverse 5’- CCCATTGTGAGTTCCATATCCATCCC-3’ |
| *Resistin (Mus)* | Forward 5’- CAAACAAGACTTCAACTCCCTG-3’ |
|  | Reverse 5’- TTTTTCTTCACGAATGTCCCAC-3’ |
| *AGT (AngII) (Mus)* | Forward 5’- GGTCTCTTTCTACCTTGGATCC-3’ |
|  | Reverse 5’- GACCTTGTGTCCATCTAGTCG-3’ |
| *IL-10 (Mus)* | Forward 5’- TTCTTTCAAACAAAGGACCAGC-3’ |
|  | Reverse 5’- GCAACCCAAGTAACCCTTAAAG-3’ |
| *Pla2g2d (Mus)* | Forward 5’- CTGTGGACTTGGTGGCAAAGGG-3’ |
|  | Reverse 5’- TCTTCAGGTGGGCATAGCAACAATC-3’ |
| *Gpr120 (Mus)* | Forward 5’- GCCTGGTGCTCAACCTCTTCTG-3’ |
|  | Reverse 5’- ACGCTGCCGCTCATTGTCATC-3’ |
| *Gapdh (Mus)* | Forward 5’- ACTCCACTCACGGCAAATTC-3’ |
|  | Reverse 5’- TCTCCATGGTGGTGAAGACA-3’ |
| *TNF-α (Rat)* | Forward 5’- CACCACGCTCTTCTGTCTACT-3’ |
|  | Reverse 5’- AGATGATCTGAGTGTGAGGGTC-3’ |
| *IL-6 (Rat)* | Forward 5’- GTTGCCTTCTTGGGACTGA-3’ |
|  | Reverse 5’- GTTGTGGGTGGTATCCTCTGT-3’ |
| *MCP-1 (Rat)* | Forward 5’- AGGACTTCAGCACCTTTGA-3’ |
|  | Reverse 5’- TTCTCTGTCATACTGGTCACTTC-3’ |
| *Adiponectin (Rat)* | Forward 5’- AATCCTGCCCAGTCATGAAG-3’ |
|  | Reverse 5’- CATCTCCTGGGTCACCCTTA-3’ |
| *Omentin (Rat)* | Forward 5’- CAGAGGGTGCCACAAGTGATGAC-3’ |
|  | Reverse 5’- GAAGCCAGTGAAGGTGCGGTAC -3’ |
| *Resistin (Rat)* | Forward 5’- ACTTCAGCTCCCTACTGCCA-3’ |
|  | Reverse 5’- GCTCAGTTCTCAATCAACCGTCC-3’ |
| *AGT (AngII) (Rat)* | Forward 5’- AGGCAAGAGGTGTAGCCAGT-3’ |
|  | Reverse 5’- AGGACCTTATGTCCGTCCAG-3’ |
| *IL-10 (Rat)* | Forward 5’- CTTTAAGGGTTACTTGGGTTGC-3’ |
|  | Reverse 5’- CCACTGCCTTGCTTTTATTC-3’ |
| *Pla2g2d (Rat)* | Forward 5’- CTGCTGGCTGGTATAACTG-3’ |
|  | Reverse 5’- GAAGGCTTTCTTCCCAGTC-3’ |
| *Gpr120 (Rat)* | Forward 5’- CCAACCGCATAGGAGAAATC-3’ |
|  | Reverse 5’- GAAGGAAACCATGAGCAGGA-3’ |
| *Gapdh (Rat)* | Forward 5’- TCTCTGCTCCTCCCTGTTCTA-3’ |
|  | Reverse 5’- ATGAAGGGGTCGTTGATGGC-3’ |
