# Supplementary figures and images for: Single-cell view and a novel protective macrophage subset in perivascular adipose tissue in T2DM
Source: Cell Mol Biol Lett. 2024 Dec 3;29:148. doi: 10.1186/s11658-024-00668-5 (PMC11616190; doi:10.1186/s11658-024-00668-5)

**Figure 4D**

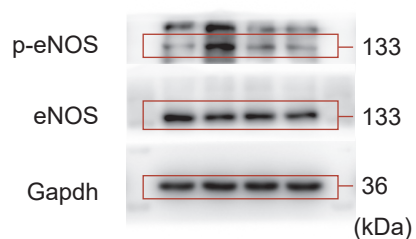

**Figure 5C**

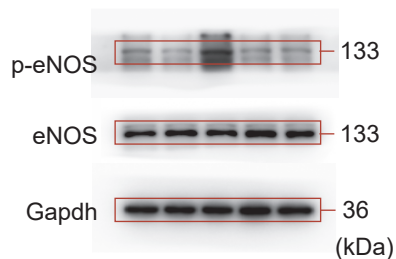

**Figure 6C**

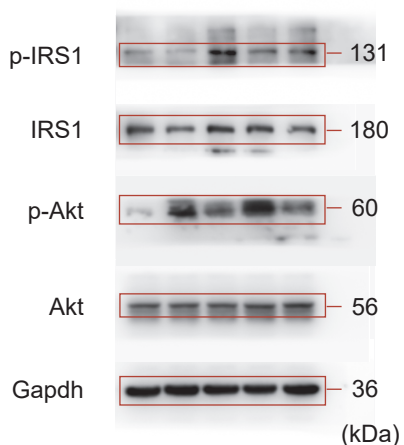

**Figure 6E**

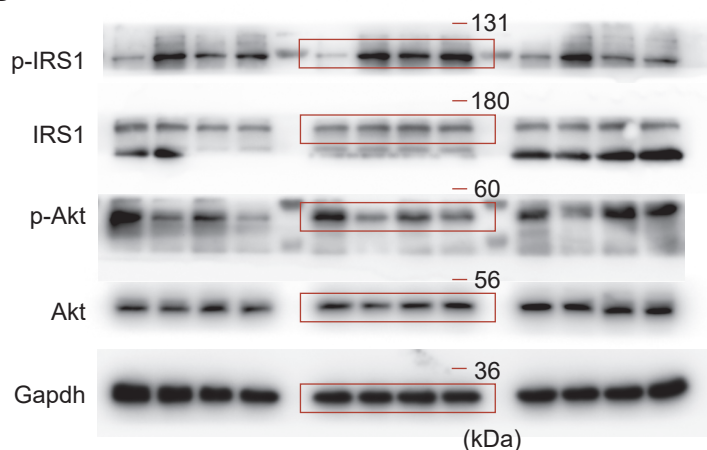

**Figure 6H**

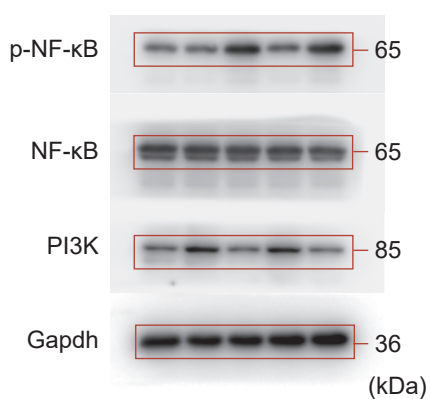

**Figure 6I**

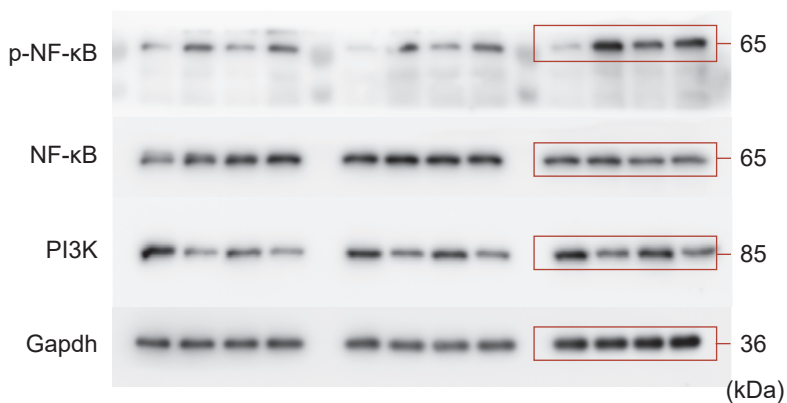

**Figure 7B**

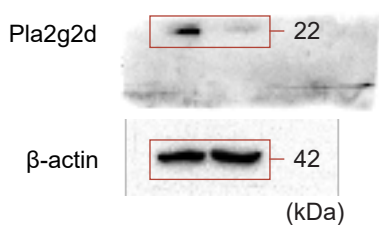

**Figure 7F**

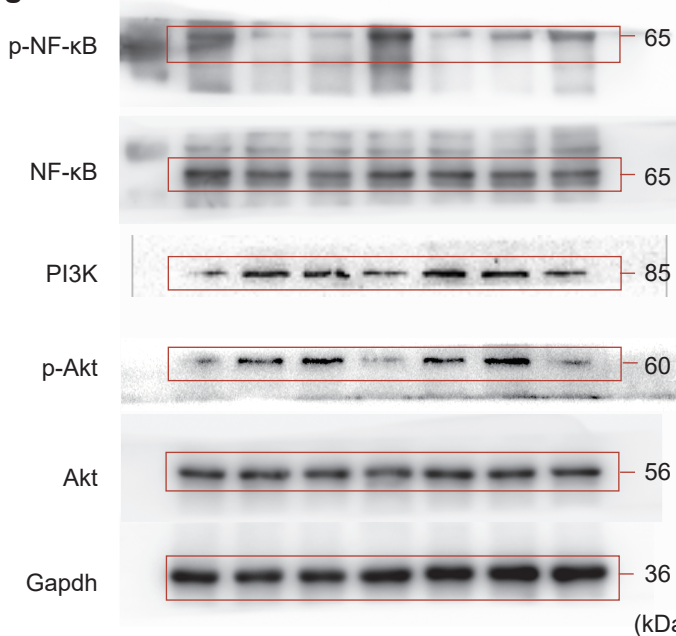

**Figure 8B**

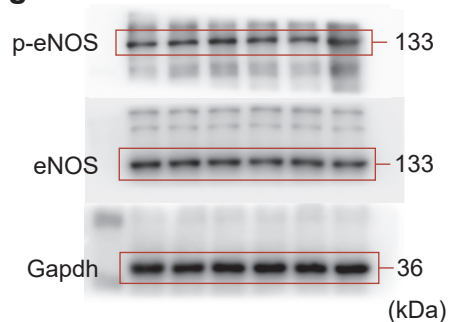

Supplement: Supplementary file 2 — Additional file 2. Original western blots. [file 11658_2024_668_MOESM2_ESM.pdf]
